# The biologics manufacturing patent notice challenge: a comparative examination

**DOI:** 10.1093/jlb/lsaf025

**Published:** 2026-08-14

**Authors:** Osmat Azzam Jefferson, Arti Rai

**Affiliations:** Senior Research Fellow, Duke Law Center for Innovation Policy, 2024–2025; now at Azzam Research Group; Duke University School of Law, Durham, NC, USA

**Keywords:** Biologics, manufacturing, patents, BPCIA, biosimilars, notice

## Abstract

In a recent study, we created and analyzed a novel dataset of US biologics manufacturing patents held by nine leading firms that produce originator therapeutic proteins. Here, we expand our data and analysis to encompass not simply US patents but also their family members, with the total set constituting a substantially broader group of patents held internationally. Our wider focus allows us to compare firm strategies in the USA and other jurisdictions. We also contrast the strategies of US-based firms with those headquartered in Europe. Unlike prior comparative studies, which focus primarily on litigated patents, we assess a broader competitive landscape. Our analysis yields several findings. First, all nine firms accumulate many more biologics patents in the USA than in jurisdictions like Europe and Japan. They do so in part by accumulating large numbers of patents with claims of varying scope over a long time period within a given US family. Second, we show distinct patterns of patent accumulation based on firm type. US-based firms with origins in biologics research and development (R&D) that continue to have internally focused R&D efforts dominate patent accumulation not only in the USA but also in Europe and Japan. In contrast, firms that make small molecules in addition to biologics secure fewer patents, regardless of their location. As a detailed illustration of patent practice, we provide a case study of Regeneron’s Eylea. Overall, our findings indicate a substantial notice challenge for biosimilar entry and provide support for reform that increases transparency and information flow regarding biologics patents generally, and manufacturing process patents specifically. Our results also support mechanisms for reducing the anti-competitive impact of large patent families, such as limitations on the number of late-filed patents within a family that can be asserted against biosimilar firms.

## I. INTRODUCTION

To increase competition and help reduce prices of costly biologic drugs, the US Congress passed the Biologics Price Competition and Innovation Act of 2010 (BPCIA). The BPCIA created a pathway for firms to seek the FDA approval for, and enter the market with, biosimilar equivalents to originator biologics. In addition, when the biosimilar manufacturer submits its initial license application to the FDA and the agency agrees to review it, the BPCIA provides a set of non-mandatory procedures for addressing patent issues. These procedures, known colloquially as the ‘patent dance,’ involve the originator and would-be biosimilar manufacturer respectively exchanging patent and manufacturing process information in a stepwise fashion. Specifically, after the biosimilar firm provides its manufacturing process information to the originator, the originator puts forward a list of patents it believes that the biosimilar infringes. The result of subsequent ‘good faith’ negotiations over this list is a list of patents that the firms agree to litigate in a ‘first round.’ Patents not litigated in the first round can be litigated in a second round, which commences when the biosimilar firm provides a required 180-day notice of commercial marketing. Litigation leads in most cases to a settlement; occasionally the case goes to a final judgment regarding patent validity and infringement.^1^

Reviewing complaints from BPCIA lawsuits, Van de Wiele et al.^2^ reported that in 90 per cent of the cases, originator firms alleged that biosimilar manufacturers did not comply with the BPCIA litigation process. Most notably for present purposes, Dong^3^ found that in all but two litigated cases, the originator manufacturers alleged that biosimilar applicants either failed to provide information regarding their manufacturing process or provided insufficient information. Biosimilar firms, particularly those that are also originators, may be concerned about protecting their own intellectual property. They might even worry that if they provide manufacturing process information, originator firms could add patent claims that cover methods or processes used by the biosimilar firms.^4^ In fact, in a prior study wherein we created a novel dataset of manufacturing process patents from nine leading originator firms, we found that seven out of the nine firms continued to file manufacturing ‘platform’ patents (ie, patents not specific to any single biologic) many years after the FDA approval of specific biologics.^5^ As we explained in that article, platform patents, which can be asserted against biosimilars of multiple different biologics, may be particularly important for blocking biosimilar entry.

However, to better understand the noncompliance issues raised under the BPCIA framework, as well as potential impediments to biosimilar entry more generally, it is important to evaluate not only platform patents but also the larger context surrounding how originator biologics firms construct and expand their US biologics patent portfolios over time, and in comparison with other jurisdictions.

Hence, in this contribution, we expand our dataset of manufacturing patents held in the USA by their simple family and analyze a substantially larger set of related and internationally held biologics patents by the nine originator firms. Prior comparative studies have shown that biologics patents are asserted more frequently in litigation in the USA than in other countries.^6^ Particularly relevant to our analysis, 53 per cent of patents litigated in the USA with no foreign equivalents have manufacturing process claims.^7^ Unlike these prior comparative studies, which focus primarily on litigated patents, we assess a broader competitive landscape, examine both domestic and international family members, and include both grants and applications.

Given our international and comparative focus, we first review patent filing flexibilities afforded by international conventions and treaties such as the Patent Cooperation Treaty (PCT) (1970) or the Paris Convention (1883). These flexibilities help firms pursue competitive advantage by building large international patent portfolios, establishing international alliances, or investing in innovations that strengthen and sustain that advantage over time. We next turn to our research methods and basic data.

### I.A. Flexibilities Afforded to International Firms

International firms have at least three major patent filing route options: (i) the national route, (ii) the national and international route, and (iii) the international then national route. For example, firms can file one or more provisional patent applications to establish an early filing date for their invention and get a 12-month period from the time of the first provisional to work on their invention before filing a non-provisional national application. In addition to a non-provisional national application, firms can also, within 1 year of their provisional application, file an international patent application under the PCT.^8^ The PCT application gives applicants 30 months from the PCT filing date to file other national applications across many jurisdictions. Advantages of the PCT filing route include both delayed second filings in national jurisdictions and cost savings. Moreover, filings in national jurisdictions can be informed by the written opinion reports issued by an International Searching Authority within 16 months after the first filings. Alternatively, within a year of filing a national application, a firm can file an international application under the 1883 Paris Convention,^9^ however, the Convention route gives applicants in any jurisdiction only 1 year to file other applications outside their original jurisdiction. Rather than starting with a national application, applicants can also first file an application through the European Patent Office (EPO) under the European Patent Convention.^10^ The European patent application can be a priority application, and if successful, it leads to a European patent granted by the EPO. However, in contrast with PCT applications, European patents are limited to Europe and must also be registered in national EPO jurisdictions.

As firms accumulate patents based on an earliest priority filing, their collections may include multiple patents within a single domestic jurisdiction. These additional domestic patents encompass divisional applications (in Europe and Japan) or continuation and divisional applications (in the USA). The resulting members of the international patent ‘family’ all benefit from the filing date of their first-filed ‘parent.’ That earliest filing date defines when prior art can be considered relevant against the application.

Whereas firms can have several types of patent families based on priority filings,^11^ in this study, we focus on simple patent families (DocDB simple patent family definition)^12^ wherein all family members share a priority date and each family represents a unique invention.

### I.B. Basic Data and Methods

Detailed methods used to construct the first manually curated manufacturing patent collection are reported in Jefferson et al. (2025).^13^ The operational definition for a manufacturing process patent requires at least one claim drawn to: (i) the preparation and production of the master cell line containing the gene for the desired protein; (ii) the large-scale growing of cells that produce the protein, along with tools designed for that purpose; and (iii) the isolation, purification, analysis, and post-production modification of the protein to make it ready for therapeutic use. We also include claims drawn to methods for producing engineered proteins and formulations. Full details about the claim categories excluded during the curation process, as well as the actual data collections, are publicly available at https://link.lens.org/6AI7Bq8WMfd.

To implement this definition, we queried the Lens platform using key terms relevant to the manufacturing process workflow of biologic products and then performed several cleanup and manual curation steps based on claim analysis. Our queries focused on nine leading firms in the originator therapeutic protein biologics. Seven of these had a 50.7 per cent biologics drugs market share globally in 2019^14^ and, in 2022, eight of them were on IQVIA’s list of the ‘top ten’ biologics manufacturers by US sales. These firms were AbbVie, Amgen, Bristol Myers (BMS), Genentech, Janssen (J&J), Merck, Novartis, and Sanofi. We excluded two firms, Novo Nordisk and Eli Lilly, whose sales are boosted by non-traditional biologics. We added Regeneron, a prominent biologics firm just outside the top 10 that has recently become quite active in litigation against biosimilars.

We used FACTSET to find, and include, most parents and subsidiaries associated with these nine firms. For AbbVie and Genentech, however, we included their parent companies (Abbott and Roche, respectively) only when these were co-assignees/co-applicants on the patent; both Abbott and Roche have vast operations outside the biologics arena. Additionally, we included only subsidiaries that appeared to be contributing to the selected firm’s biologics products.

We retrieved both manufacturing platform patents applicable to more than one biological product as well as patents with claims specific to single biologics. Because our queries focused on retrieving manufacturing platform patents, our dataset may be underinclusive with respect to biologic-specific patents and other patent categories. That said, 34.8 per cent of our dataset encompasses biologic-specific patents.

Each firm’s final Lens collection, filtered by date range (dates of actual filing between 2004 and 2022), was expanded by the DocDB simple family definition in the Lens platform and downloaded on Nov. 5, 2024. A non-redundant set for each of the USA and the international family members was then manually created to examine and analyze their structure and distribution (Table 1).

**Table 1:** Counts of manually curated US manufacturing process patents, their corresponding simple families, and the international family members accounted for in the Lens platform.

Firm	US manufacturing process patents	Corresponding simple family count ^a^	International family members (expanded by simple family) ^b^
Regeneron	236	107	4522
Genentech	136	95	3133
Amgen	128	80	2200
Merck	108	83	1231
AbbVie	86	53	1301
Janssen	72	56	1245
BMS	71	55	1211
Novartis	67	51	1099
Sanofi	61	38	1274
Total	965	618	17,216

^a^Simple family counts in the Lens platform. Comparable family counts were obtained in Orbit Intelligence platform.

^b^This is a non-redundant count of both grants and patent applications available on Nov. 5, 2024.

The non-redundant sets all encompass families with at least one US manufacturing process patent member. Notably, however, the expanded members of each family may include not only patents drawn to manufacturing processes, but also grants or applications with claims drawn to composition of matter as well as methods of use, treatment, or delivery (dosage, injection, or formulation). The expansion thereby allows us to assess decisions to file manufacturing patents relative to other types of patents. All constructed datasets are available for noncommercial use.

## II. DATA ANALYSIS

### I‌I.A. Dominance of the Originator Biologics Firms’ US Patent Family Members

We began our data analysis by examining the composition of international patent families. To do this, we extracted the family members for each firm from the Lens platform, along with various attributes including claims. Given the unique continuation feature of US practice that enables firms to increase family size and filing timespans in ways that are not possible under the European or Japanese systems, we then proceeded to manually build a separate non-redundant US subset and computed the domestic family size (number of US patent and patent application family members) and the filing timespans (time between the first filed application and the last filing within a family).

A simple comparison of the total US patent family collection with those from the EPO (EP) or Japan (JP) shows that all firms have a higher percentage of US family members in their international patent portfolios (Table 2). For example, 30 per cent of the BMS international patent portfolio consists of US patent members compared to 11 per cent EP members and 8 per cent Japanese members, respectively.

**Table 2:** The percent of USPTO, EPO, and JPO patent family members (grants and applications).

A-Firm	% of US family members ^a^	% of EP family members	% of JP family members
BMS	30%	11%	8%
AbbVie	28%	8%	7%
Merck	23%	13%	11%
Amgen	18%	10%	9%
Janssen	18%	12%	8%
Novartis	18%	12%	9%
Regeneron	18%	8%	9%
Genentech	17%	9%	9%
Sanofi	17%	11%	7%

^a^The percentage of family members includes both grants and applications as well as those with no foreign equivalents.

As for family size and filing timespans, the data from the US subset show wide differences within and across firms (Table 3). Across firms, the maximum family size range was between 15 (Janssen) and 65 (Regeneron) with the average ranging between 3.4 and 7.5 (median range between 2 and 5). Within a firm, the max−min gap size was also consistently large. As for the filing timespans, across firms, the maximum timespans was slightly narrower and ranged between 14 (Sanofi) and 19.8 years (BMS). However, there were substantial gaps between the min−max, average, and median filing timespans.

**Table 3:** Min, max, average, and median of US family size and family timespans.

B-Firm	Max of US family size	Min of US family size	Average of US family size	Median of US family size	Max of filing timespans	Min of filing timespans	Average of filing timespans	Median of filing timespans
Regeneron	65	2	7.5	5.0	19.1	1.0	7.1	6.3
AbbVie	59	2	6.9	4.0	15.7	1.0	5.5	5.2
Sanofi	42	2	5.8	4.0	14.0	1.0	6.6	7.2
Genentech	26	2	5.6	4.0	19.0	1.0	8.3	7.5
BMS	25	2	6.6	4.0	19.8	1.0	7.6	7.2
Novartis	20	1	3.9	3.0	15.3	0.0	5.1	4.8
Merck	18	1	3.4	2.0	16.3	0.0	3.2	1.5
Amgen	16	1	4.9	4	19.2	1	6.7	6
Janssen	15	2	4.0	3.0	15.5	0.0	4.8	4.1

^a^Family size includes both grants and applications.

^b^Filing timespans was calculated by days and then converted to years.

In summary, our results demonstrate that all nine originator firms hold larger US patent portfolios relative to portfolios held in Europe or Japan. Regeneron, AbbVie, and Sanofi have the largest US family sizes, which range between 42 and 65 members. The family size distribution is asymmetric, as all firms have a higher family size average than median. For the filing timespans, the data also show that all nine firms extend their filing timespans substantially, to a maximum range between 14 and 19.8 years from the first filing. However, the distribution is also skewed, likely due to a significantly different value for some patent families or even selected patent members within a family.

The selective practice of extending the US patent filing timespans by many years, which can enable an originator firm with a pending patent application to add or change the scope of its claims to cover competitors’ products, may explain the previously reported reluctance of would-be biosimilar entrants to share their manufacturing process information during the initial disclosure phase of the BPCIA patent dance.^15^

More generally, from the standpoint of the would-be biosimilar entrant, the information exchange set up by the BPCIA is asymmetric. Asymmetry arises because while the BPCIA provides a mechanism by which originator biologics firms can, at least in principle, secure access to biosimilar manufacturing information, biosimilar firms seeking to show the invalidity of an originator manufacturing patent must engage in time-consuming litigation discovery procedures to obtain information regarding the process that the originator used at approval. Perhaps not surprisingly, the majority of cases settle before judicial determinations of validity.

The BPCIA also fails to provide would-be biosimilar firms with *timely* notice of relevant patents. Although originators have, since the Purple Book Continuity Act of 2020 (‘Continuity Act’) been required to list some patent information, this listing occurs late in the game, only after a first biosimilar firm files its FDA application and commences the patent dance.^16^ Although this dearth of notice is a problem for all patents, it may be particularly acute for manufacturing process patents. While a biosimilar firm may, through examination of the originator biologic product at approval, be able to determine whether its patent searches have identified patents that cover the product at launch, the launched product does not reveal the process by which it was made.

### I‌I.B. Firm Type Affects Patterns of Patent Accumulation

The nine firms we studied vary by headquarters (Switzerland for Novartis, France for Sanofi, and the USA for the other seven) and by whether they focus primarily on biologics (Abbvie, Amgen, Genentech, and Regeneron do, while the other five do not). Accordingly, we could test for patterns of patent accumulation by firm type.

Following the Dechezleprêtre et al. (2017)^17^ methodology, we first determined the earliest priority filing for each of the firms’ US manufacturing patent members and then mapped their other family members based on the following categories: (i) domestic patent members of an international family with a *domestic* priority, (ii) domestic patent members of an international family with a *foreign* priority, and (iii) domestic patent members of a national family only. Next, we calculated the distribution of family members per country of priority. Table 4 summarizes the results for all firms based on the USA, Europe, and Japan as countries of priority.

**Table 4:** Distribution of biologics patent family members in the international dataset of each firm and according to the domestic or foreign priority filings. Countries of priority include the USA, Europe, and Japan.

Country of priority	Firm	Domestic members of international families with domestic priority	Domestic members of international families with foreign priority	Domestic members of domestic (single office) families ^a^	Total family members per country
USA	Genentech	96%	0%	4%	527
	Regeneron	89%	0%	11%	815
	AbbVie	84%	4%	12%	367
	Amgen	83%	3%	14%	392
	Janssen	65%	22%	13%	222
	BMS	63%	9%	28%	361
	Merck	59%	29%	12%	280
	Novartis	53%	45%	1%	201
	Sanofi	27%	62%	11%	221
Europe	Genentech	0%	100%	0%	297
	Regeneron	0%	100%	0%	341
	AbbVie	3%	97%	0%	104
	Amgen	4%	84%	0%	213
	BMS	8%	92%	0%	134
	Janssen	15%	85%	0%	150
	Merck	43%	57%	0%	166
	Novartis	51%	49%	0%	127
	Sanofi	82%	18%	0%	146
Japan	Genentech	0%	100%	0%	291
	Regeneron	0%	100%	0%	408
	AbbVie	0%	100%	0%	90
	Amgen	0%	100%	0%	190
	Janssen	0%	100%	0%	104
	Novartis	0%	100%	0%	104
	Sanofi	0%	100%	0%	92
	Merck	2%	98%	0%	132
	BMS	4%	96%	0%	95

^a^Amgen, Merck, Novartis, and Regeneron each have one US family with a single patent member but these were excluded from this column. Only families with more than one member were accounted for.

Looking at the USA as the country of priority, we found that eight out of the nine leading biologics manufacturers have a range of their US patent family members (11−28 per cent) with no foreign equivalents, compared to Europe or Japan, which had none. The exception, Switzerland-based Novartis, had only 1 per cent of its US patent family members with no foreign equivalents. In addition, none of Regeneron’s and Genentech’s US patent family members had a foreign priority; similarly, none of Regeneron’s and Genentech’s European and Japanese filings had a domestic priority. In contrast, Novartis and Sanofi respectively had 45 per cent and 62 per cent of their US members with foreign priority filings.

The distinct filings and differential patent accumulation patterns suggest that US-based originator firms with internally established research and manufacturing capabilities in biologics, such as AbbVie, Amgen, Genentech, and Regeneron, operate more independently and may have different patenting strategies compared to the other five firms, which may have alliances and subsidiaries in various countries.

### I‌I.C. Levers for Constructing US Patent Estates

To construct a more detailed map of the levers firms use to build their US patent estates, we selected 8−10 families per firm and focused our analysis on the US issued patent members within each family. Family selection was based on the descending order of the number of the US issued patent members, which ranged in the subset from 3 to 31, with Regeneron having the highest number, followed by AbbVie and BMS (Supplementary Table S1, Appendix A). Within each of these families, we initially examined the filing route of the first issued US patent member, tracked chronologically all its related publications including original USA and PCT applications based on the filing date, manually added the continuation types of second filings, and checked the claims. In total, we assessed 88 US domestic families representing all nine leading originator biologics manufacturers.

#### I‌I.C.1. Flexible filing routes

**Figure 1:**
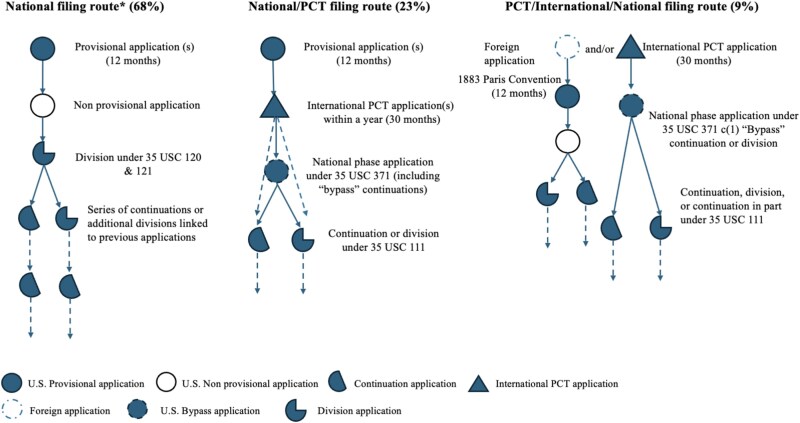
Filing routes of the selected 88 US patent families from nine leading biologics manufacturers. ^*^Applicants using this route generally also obtained international protection. All constructed datasets are available for noncommercial use.

Six out of the nine firms used both the national and the combined national/PCT filing routes to file their first US issued patent member. The remaining three (Janssen, Novartis and Sanofi) used all three routes. The computed ratio of route usage indicated that 68 per cent of these domestic families used the US-only filing route, 23 per cent used both the USA and PCT routes, and 9 per cent used the international/PCT/national filing route (Fig. 1). The last group of families was mainly from Novartis and Sanofi, which had foreign application priority data. Upon further analysis of families with national filing routes, we found that 60 per cent of these had a single US provisional application as the initial first filing, but 27 per cent had a range between 2 and 5 provisional applications (Supplementary Table S2, Appendix A). In the USA, provisional applications combined with the use of continuations and divisionals, allow firms to delay second filings and/or extend the filing timespans. In this case, our data show that all originator firms were able to delay their second filings by 5−9 years, with Amgen and Genentech having the longest delay.

#### I‌I.C.2. Tailoring the scope of claims within an invention and over time

Examining the claims of the issued patents in the 88 families revealed a consistent and expansive claiming strategy adopted by the nine firms. Within a single family (one invention) and sometimes under the same title and abstract, the applicant(s) have multiple US patent family members with claims of varying scope. This lever is facilitated by the unique US feature of continuation practice, which allows patent applicants to maintain applications in a pending prosecution state over a long period of time so that they can add to or change the scope of claims.

In Appendix B, we present a family example from each of the nine firms to demonstrate the comparable claiming strategies adopted by these firms.

#### I‌I.C.3. Securing secondary patents and patenting dosage changes within the BPCIA regulatory exclusivity period

Following the market entry of an originator product, the BPCIA gives the originator firm a 12-year data exclusivity period. Moreover, under Section 505A of the Food, Drug, and Cosmetic Act, the FDA can offer an additional 6 months of pediatric exclusivity if the firm agrees to conduct pediatric studies. During this period, biosimilar firms cannot rely on originator data to launch, but an originator firm can continue developing its product, patent newly improved manufacturing processes, formulations, or packaging, and seek approval on new indications. Around the time the exclusivity period is slated to end, the firm can switch to a new version that is protected by additional patents – a phenomenon known as ‘product hopping’.^18^ Alternatively, instead of an abrupt switch, the firm can secure approval for secondary changes and gradually move the market to the new version, thereby reducing the market size for biosimilars of the old version.

To illustrate the practice of all three levers by a leading US originator biologics firm with internally focused research and manufacturing capabilities, in Section II.D, we zoom in on Regeneron as a case study. We analyze two Regeneron patent families that are relevant to its first human fusion protein product, Eylea (aflibercept), and map the changes that occurred prior to and since its approval and market launch.

### I‌I.D. Case Study: Regeneron

The first patent family (Family 49) involves Regeneron’s original Eylea product, has 13 US issued patent members with no foreign equivalents, and is extended over 18 years. The family began as a US provisional application No. 60/138,133, filed on June 8, 1999, then a PCT application filed on May 23, 2000, followed by bypass divisionals/continuations until 2017. As shown in Supplementary Table S3 (Appendix A), the first US patent family member, filed in 2001, had composition of matter claims for an isolated nucleic acid encoding a fusion protein capable of binding vascular endothelial growth factor (VEGF), its sequence, an expression vector comprising that molecule, and a host-vector system for the production of that protein. The second and third patents, filed ‘3 years later,’ covered more details about the fusion protein, its modified sequence, and treatment methods using that protein. Three subsequent patents, ‘filed three and a half years later,’ covered variants of such proteins and modified treatment methods. By 2011, when Regeneron submitted and received the FDA approval for its original product, Eylea, the firm had two additional patent family members on the host cell containing the expression vector to express the protein and its variants, and a cell culture method patent to express that protein. From 2012 to 2017, subsequent patents were published on a method of producing the fusion protein (a manufacturing patent), characteristics of the chimeric protein, and a method for making a nucleic acid molecule encoding a chimeric VEGF-binding fusion protein (another manufacturing patent). During that period, Regeneron continued to also receive the FDA approval for additional new Eylea indications.

Under Biological License Application (BLA) 125,387, the original Eylea (2 mg) was approved on Nov. 18, 2011, for the treatment of neovascular (wet) age-related macular degeneration (AMD). Since, the following six indications have been approved: (i) treatment of secondary macular edema (S004) on Sep. 21, 2012, (ii) treatment of diabetic macular edema (DME) (S037) on July 29, 2014, (iii) treatment of secondary macular edema (S043) on Oct. 6, 2014, (iv) treatment of diabetic retinopathy in patients with DME (S048) on Mar. 25, 2015, (v) treatment of diabetic retinopathy (S061), on May 13, 2019, and (vi) treatment of retinopathy of prematurity (S075) on Feb. 8, 2023.

Along with these approvals, Regeneron has continued to file patents on various aspects of manufacturing Eylea products. Five years before the expiry of Eylea’s regulatory exclusivity, Regeneron filed two new US provisional applications: No. 62/944,635, filed on Dec. 6, 2019, and No. 63/065,012, filed on Aug. 13, 2020, which led to six original patents, succeeded by a series of divisionals/continuations until June 2023. The first few patents (marking the beginning of the second family example, Family 18, as shown in Supplementary Table S4, Appendix A) claim improved methods of producing aflibercept and/or a modified version of it. Thus, the family involves a variation of the original product and now, through subsequent continuations and divisionals, has 65 family members, 31 of which are US-issued patents with claims on various aspects of manufacturing and delivering Eylea and its variants^19^ over the span of 5-year period. The family has a priority date of Dec. 6, 2019, 8 years *after* the FDA approval of Eylea, and highlights the complex connections between regulatory exclusivities available under the BPCIA and the various other levers deployed to extend and expand market control.

Below, we map the specific changes that led to the approval of Regeneron’s new High Dose Eylea product on Aug. 18, 2023, 3 months before its reference product exclusivity expiration date of Nov. 18, 2023, and 9 months before its pediatric exclusivity expiration date of May 18, 2024, and discuss their influence on market dominance and domestic biosimilar competition.

In December 2022, based on the FDA permission for a ‘rearrangement of dose,’ Regeneron filed a new BLA (BLA 761355) to register a high dose of its original Eylea (Eylea HD). As suggested by Regeneron, the FDA also accepted cross-referencing to the clinical studies that Regeneron had already been conducting for neovascular AMD (nAMD) and DME under the original EYLEA BLA (125387), for the registration of these two indications for Eylea HD. Consequently, the HD submission received a priority review, and the FDA approved the alternative dose regimen of Eylea HD for the treatment of nAMD and DME. Eylea HD is basically an 8 mg instead of a 2 mg dose of aflibercept administered intravitreally in intervals of up to 16 weeks, instead of 2 mg of the same drug administered every 8 weeks.

#### II.D.1. Securing Eylea dominance in the market

Regeneron (in the USA) and Bayer (in Europe) are jointly developing Eylea HD, and their recent 3-year clinical trial results show that patients who switched from the Eylea 2 mg injection to Eylea 8 mg seemed able to maintain vision and anatomic improvements through the end of the third-year trial, but with fewer injections and longer dosing intervals.^20^ In both the USA and Europe, Eylea HD is the only treatment that is approved for extended treatment intervals of up to 5 months in wet AMD and DME. On Feb. 10, 2025, Bayer submitted a new application to the European Medicines Agency to expand treatment intervals to up to 6 months in both diseases and on June 27, 2025, the European Commission approved it in the European Union.^21^

Following the FDA approval of Eylea HD, Regeneron announced its US list price would be $2625.00 per single-use vial with the anticipation that the annualized list price of EYLEA HD would be in ‘the range of, or lower than, EYLEA’.^22^ In 2024, net sales for EYLEA HD and EYLEA combined were $5.97 billion, with $1.20 billion from EYLEA HD.

The continued development of Eylea HD, along with the noticeable progress in extended treatment trials and the increase in net sales of Eylea HD in 2024, points to a gradual shift to Eylea 8 mg from Eylea 2 mg. Regeneron maintains exclusive rights to both the original Eylea and Eylea HD in the USA, whereas Bayer holds an exclusive marketing license outside the USA. Both companies will share profits from the sales of both products equally.^23^

In Section II.D.2, we argue that the combination of the 12-year exclusivity afforded to Regeneron and the firm’s carefully tailored US patenting strategy has enabled it to assert patents filed as late as 11 years after the FDA approval of the original Eylea product against six biosimilars of this product, and, at least for now, forestall competition against five of them. Meanwhile, as we have discussed, the market appears to be shifting to Eylea HD.

#### II.D.2. Regeneron and US biosimilar entry

To assess the impact of Regeneron’s multi-layered use of its US patenting strategy on entry by biosimilars of original Eylea, we downloaded BPCIA complaints filed by Regeneron against six biosimilar applicants [Mylan/Biocon (Viatris), Celltrion, Samsung Bioepis, Formycon, Amgen, and Sandoz] from the Goodwin website,^24^ analyzed them, and extracted manually each of the patent lists that Regeneron had asserted during the BPCIA patent dance. We then matched these to our US expanded family biologics dataset for Regeneron, checked the matched patents’ attributes including their filing dates and patent type, and grouped them by family for further analysis.

Supplementary Table S5 (Appendix C) shows the analyzed BPCIA complaints, and a summary of the allegations made along with other relevant contextual information. On average, Regeneron has asserted 35 patents per complaint (the range is between 24 and 46 patents). Between 52 and 64 per cent of Regeneron’s asserted patents matched patents in our collection, and several of these patents were asserted against two or more applicants (Supplementary Table S6, Appendix C). This match percentage is quite high given that we excluded patents with independent claim categories drawn to methods of treatment, methods of use, and delivery (dosage, formulation, or packaging) in our initial manually curated manufacturing platform patent collection. For example, two frequently asserted but unmatched patents in our dataset were patent No. U.S. 11,084,865 (issued on Aug. 10, 2021) and 11,793,926 (issued on Oct. 24, 2023) which cover formulation and packaging-related claims, respectively.

Removing redundant patents and grouping the matched patents by family revealed the assertion of 37 unique Regeneron patents, representing 12 distinct families. Within a single family, several patent members have been asserted over time. For example, in family 18 (discussed above) 16 out of the 31 issued patents were asserted at different times and in various combinations against biosimilar applicants. By tracking the filing dates of such patents, we found that Regeneron has asserted patents filed as many as 11 years after the FDA approval date of Eylea in 2011 and that 78 per cent of its asserted patents are second filings (continuations and/or divisionals).

Notably, despite the expiration of original Eylea’s regulatory exclusivity on May 18, 2024, and the FDA’s approvals of Eylea biosimilars from Mylan/Biocon (Viatris), Samsung Bioepis, Formycon, and Sandoz, none of these firms has yet been able to market launch their Eylea biosimilar in the USA. On June 14, 2024, a district court granted Regeneron’s motions for a permanent injunction against Mylan/Biocon and a preliminary injunction against Celltrion, Samsung Bioepis, and Formycon. These determinations were affirmed by the Federal Circuit upon Jan. 29, 2025.^25^ However, the district court, and recently the Court of Appeals for the Federal Circuit affirmed the District Court’s denial of a preliminary injunction against Amgen, which decided to launch its Eylea biosimilar Pavblu (aflibercept-ayyh) on Nov. 5, 2024.^26^

Amgen’s launch price for Pavblu is $1664 per injection, which is only 10 per cent less than that of a single dose of Eylea’s 2 mg ($1896.25). Experts predict that such entry is unlikely to have a significant market effect on Eylea, as other lower cost options, such as the use of Vabysmo, the off-label Avastin, and the Lucentis biosimilars, are currently available. In addition, as noted, Pavblu faces market competition from products with less frequent dosing such as Eylea HD.^27^

In Europe, Bayer AG—the exclusive licensee of Regeneron outside the USA—has held a market authorization in the European Union on Eylea (2 mg) since November 2012 and Eylea (8 mg) since January 2024. But from September 2023, the European Commission has authorized the use of seven Eylea biosimilars; Eydenzelt (Celltrion Healthcare Hungary Kft.), Pavblu (Amgen Technology (Ireland) UC) Yesafili (Biosimilar Collaborations Ireland Limited), Opuviz (Samsung Bioepis NL B.V.), Afqlir (Sandoz GmbH), Ahzantive (Klinge BioPharma), and Baiama (Formycon AG).^28^ Following the launch of Eylea 8 mg with extended treatment intervals, Bayer registered sales growth in Europe from €3231 million in 2023 to €3306 million in 2024,^29^ and the recent approvals of extended treatment intervals of up to 6 months in Europe and other countries signal a steady move to gradually shift to the new product in Europe as well.

## III. DISCUSSION

Our review of the international patent landscape of nine leading originator firms indicates that their US manufacturing process patent estates are substantially more extensive than their estates in other leading jurisdictions. Moreover, we show distinct patterns of patent accumulation based on firm type. US-based firms that came into existence producing biologics and continue to have internally focused research and development efforts, dominate patent accumulation not only in the USA but also in Europe and Japan.

Further analysis of these firms’ US patent family portfolios reveals three major levers these firms have used to build and expand their domestic patent estates: the use of flexible filing routes, unique continuation features that allow them to extend family size and filing timespans, and the ability to secure secondary patents during the 12-year regulatory exclusivity period available after the market launch of their original product. To further demonstrate these practices, we provide a detailed case study of Regeneron.

This case study demonstrates in detail the substantial contrast between the USA and Europe vis-à-vis biosimilar entry. Through the combined use of its domestic patent holdings and litigation hurdles under the BPCIA, the firm has managed to delay/deter Eylea biosimilar entry domestically. The only Eylea biosimilar that has launched is the one from Amgen. By contrast, the European Commission started authorizing Eylea biosimilars in September 2023 and to date, seven have been approved and launched. Given the recency of Eylea-related biosimilar development, the relative market impact in Europe and the USA is still difficult to gauge. However, a broad comparison of the impact of biosimilar competition in the USA and Europe is revealing. In Europe between 2013 and 2024, the cumulative savings from list prices were €56 Billion (equivalent to $65 Bn USD) and the cumulative treatment days savings were €6.9 Bn ($8 Bn USD).^30^

In the USA, such figures were substantially lower. The total reported US savings since the launch of the first biosimilar (2015−2024) was $36 Bn USD and the savings in total patient therapy days was only $2.7 Bn USD.^31^

These results and our findings from nine leading therapeutic protein biologics firms provide support for several patent-related interventions that would increase transparency in the BPCIA process and boost biosimilar competition in the USA. Although our data are obviously limited to large firms and markets, and hence our reform proposals do not necessarily extrapolate to all biologics firms and markets, they do apply to a quantitatively substantial proportion of the US biologics market. Equally important, firms and products that command large markets represent the arena where changes in patent policy can actually make a difference. By contrast, regardless of patent policy, smaller markets may not be sufficiently lucrative to attract biosimilar development.^32^

On the reform front, our data support requiring at least large originators to list, ‘at the time of approval’ those manufacturing process patents—as well as other patents—they believe cover the product as approved. Various commentators^33^ have argued for this reform, which would better align litigation procedures for biologics with those currently used under the Hatch-Waxman Act for small molecules. At least for firms and products with large markets, our results demonstrate the magnitude of the need in the manufacturing process arena, where trade secrecy limits insight into what methods are being used at what time by the originator.

Because of large originator firms’ extensive use of continuation practice, this list may have to be updated to include patents that are filed after approval but nonetheless can legitimately cover the drug at approval because they have priority dates earlier than 1 year after commercial launch. However, under standard patent law governing ‘on sale’ prior art,^34^ no patent on this list could have a priority date more than 1 year after commercial launch.

Patent assertions by large originators against biosimilars should, in the first instance, be restricted to the patents on this list. Moreover, the existence of such a list would supersede many of the complicated provisions of the current BPCIA patent dance. One aspect of the dance that should be retained involves the current requirement that biosimilars provide to originators (under an assurance of restricted availability) their manufacturing process information. In order for originators to determine in an efficient fashion which, if any, of the originator’s manufacturing process patents the biosimilar is infringing, the originator needs to know this information. The provision of such information could be made mandatory. At the same time, to ensure adequate deterrence against breaches of confidentiality restrictions by the originator, sanctions for such breaches might need to be increased.

Even with a listing requirement, however, the heavy prevalence of continuation practices could undermine notice goals. Accordingly, strict limits on the listing of late-filed continuation patents – perhaps stricter than the caps on the assertion of late-filed patents currently in the proposed Affordable Prescriptions for Patients legislation^35^^,^^36^^,^^37^^,^^38^^,^^39^—are also merited.

Although an originator firm would be presumptively limited to patents on its list (and some modest number of related continuation patents), contexts might arise in which, subsequent to its receipt of the biosimilar’s manufacturing process information, the originator determined that the biosimilar infringed a patent with a priority filing date of more than 1 year after the FDA approval. This situation might arise, for example, if the biosimilar firm decided to avail itself of patented originator technology that was novel and nonobvious over the technology used by the originator at approval. To ensure such novelty and non-obviousness, however, the originator would be required to confidentially provide to the biosimilar the manufacturing process it used at approval. Moreover, the originator manufacturer would be required to certify whether or not it was using the late-patented process. As with caps on the assertion of late-filed continuations, strict limits on the assertion of processes that are not used to manufacture the reference product are merited. Again, although the Affordable Prescriptions for Patients Act proposes to cap assertions of manufacturing process patents that are not used, stricter limits are likely necessary.

Ideally, the confidential provision of ‘at approval’ process information would be required not only *ex post* but also to the patent examiner *ex ante,* Administrative agency procedures that allowed patent examiners to have confidential access to the information necessary to determine novelty and nonobviousness could thwart invalid patents at the outset, obviating the need for expensive district court litigation.

## Supplementary Material

supplementary_materials_lsaf025

## Footnotes

1 S. S. Tu, R. Goode, M. Turner and V. Van de Wiele. *Accelerating Biosimilar Market Access: The Case for Allowing Earlier Standing*, 12 J. Law  Biosci. 1–16 (2025).

2 S. Tu, V. L. Van de Wiele, A. S. Kesselheim and A. Sarpatwari. *Barriers To US Biosimilar Market Growth: Lessons From Biosimilar Patent Litigation*. 40 Health  Affairs 1198–1205 (2021). https://doi.org/10.1377/hlthaff.2020.02484.

3 Yun Dong. *Keep On Dancing: The Success And Failures Of Patent Dance As Shown By BPCIA Litigation Cases Filed After Sandoz v. Amgen*. 83 Univ. Pitt. L. Rev. Online (2022). https://doi.org/10.5195/lawreview.2022.874.

4
*Supra* note 2.

5 O.A. Jefferson, N. Price, S. Vishnubhakat, S. Tu and A. Rai. *The Puzzle of Biologics Manufacturing Platform Patents and Their Influence on Current U.S. Health Care Policies*. 43 Nat. Biotechnol. 295–299 (2025). https://www.nature.com/articles/s41587-025-02579-y.

6 R. Goode and B. Chao. *Biological Patent Thickets and Delayed Access to Biosimilars, An American Problem*. 9 J. Law  Biosci*.* lsac022 (2022). https://doi.org/10.1093/jlb/lsac022.

7 V. L. Van de Wiele, R. F. Beall, A. S. Kesselheim, and A. Sarpatwari. *The Characteristics of Patents Impacting Availability of Biosimilars*. 40 Nat. Biotechnol. 22–25 (2022). https://www.nature.com/articles/s41587-021-01170-5.

8
https://www.wipo.int/pct/en/ (last accessed September 19, 2025).

9
https://www.wipo.int/treaties/en/ip/paris/ (last accessed September 19, 2025).

10
https://www.epo.org/en/legal/epc (last accessed September 19, 2025).

11 C. Martinez. Insight into Different Types of Patent Families. Statistical Analysis of Science, Technology and Industry Working Paper 2010/2. (2010): OECD Publishing.

12
https://www.epo.org/en/searching-for-patents/helpful-resources/first-time-here/patent-families/docdb (last accessed September 19, 2025).

13
*Supra* note 5.

14
https://www.statista.com/statistics/309463/biotechnology-prescription-drug-market-share-by-top-companies-globally/ (last accessed September 19, 2025).

15
*Supra* note 3.

16 R. Feldman. *Trade Secrets in Biologic Medicine: The Boundary with Patents*, 24 Colum. Sci. Tech. L.Rev. 1 (2022). https://repository.uclawsf.edu/faculty_scholarship/1968.

17 A. Dechezleprêtre, Y. Ménière, and M. Mohnen. *International Patent Families: From Application Strategies to Statistical Indicators*. 111 Scientometrics 793–828 (2017). https://doi.org/10.1007/s11192-017-2311-4.

18 A. K. Rai, R. E. Sachs, and W.N. Price. *Cryptic Patent Reform Through the Inflation Reduction Act Harvard*. 37 J. Law  Technol. 57–104 (2023). https://jolt.law.harvard.edu/assets/articlePDFs/v37/2-Rai-Sachs-Price-Cryptic-Patent-Reform.pdf.

19 Family 18 issued patent members can also be accessed at https://www.lens.org/lens/search/patent/list?collectionId=231851 (last accessed September 19, 2025).

20
https://investor.regeneron.com/news-releases/news-release-details/three-year-results-eylea-hdr-aflibercept-injection-8-mg-patients (last accessed September 19, 2025).

21
https://europe.ophthalmologytimes.com/view/european-commission-grants-label-extension-for-eylea-8-mg-with-extended-6-month-treatment-interval (last accessed September 19, 2025).

22
https://investor.regeneron.com/static-files/146d7101-2db1-4d35-b6d0-b9b94b11c6fb.

23
https://investor.regeneron.com/news-releases/news-release-details/three-year-results-eylea-hdr-aflibercept-injection-8-mg-patients (last accessed September 19, 2025).

24
https://www.bigmoleculewatch.com/bpcia-patent-litigations/) (accessed Jan. 20, 2025).

25
https://www.cafc.uscourts.gov/opinions-orders/24-1965.OPINION.1-29-2025_2459289.pdf.

26
https://www.bigmoleculewatch.com/wp-content/uploads/sites/2/2025/03/Regeneron-v.-Amgen-DI-498-1-24-md-03103.pdf (last accessed September 19, 2025).

27
https://aishealth.mmitnetwork.com/blogs/spotlight-on-market-access/even-at-10-discount-eylea-biosimilar-pavblu-offers-lower-cost-option (last accessed September 19, 2025).

28
https://www.ema.europa.eu/en/search?f%5B0%5D=ema_search_entity_is_document%3ADocument&search_api_fulltext=eylea%20biosimilar, https://eyewire.news/news/eylea-8-mg-approved-in-eu?c4src=article:infinite-scroll (last accessed September 19, 2025).

29
https://www.bayer.com/sites/default/files/2025-03/bayer-annual-report-2024.pdf.

30
https://www.iqvia.com/-/media/iqvia/pdfs/library/white-papers/the-impact-of-biosimilar-competition-in-europe-2024.pdf (last accessed September 19, 2025).

31
https://biosimilarscouncil.org/resource/2024-us-generic-biosimilar-savings-report/ (last accessed September 19, 2025).

32 This latter problem of the ‘biosimilar void’ is outside the scope of this paper but has been addressed elsewhere. https://www.iqvia.com/Insights/The-IQVIA-Institute/Reports-and-Publications/Reports/Assessing-the-Biosimilar-Void-in-the-US (last accessed September 19, 2025).

33 B. S. Walsh, J.J. Darrow and A. S. Kesselheim. *Recent Orange and Purple Book Legislation Suggests a Need to Bridge Drug and Biologic Patent Regimes*. 40 Nat  Biotechnol 167–169 (2022). https://doi.org/10.1038/s41587-021-01204-y; Feldman, R. *Dance of the Biologics*. 39 Berkeley  Technol. Law J. 841(2024). https://lawcat.berkeley.edu/record/1300456?v=pdf; R. Feldman. *Paucity of Intellectual Property Rights Information in the US Biologics System a Decade After Passage of the Biosimilars Act*, 21 PLoS Medicine (2024b). https://doi.org/10.1371/journal.pmed.1004381.

34
*Supra* note 5.

35
https://www.congress.gov/bill/119th-congress/senatebill/1041 (last accessed September 19, 2025).

36 A. K. Rai and W.N. Price. *An Administrative Fix for Manufacturing Process Patent Thickets*. 39 Nat. Biotechnol. 20–22 (2021). https://doi.org/10.1038/s41587-020-00780-9.

37 C. Righi, D. Cannito, and T. Vladasel. *Continuing Patent Applications at the USPTO*. 52 Res. Policy 1-16 (2023). https://doi.org/10.1016/j.respol.2023.104742.

38
*Regeneron Pharmaceuticals, Inc. v. Samsung Bioepis Co., Ltd.*  https://www.cafc.uscourts.gov/opinions-orders/24-1965.OPINION.1-29-2025_2459289.pdf (accessed Jan. 29, 2025).

39
*Sandoz Inc v. Amgen Inc*, 582 U.S. 1 (2017).

