# Supplementary material for: The biologics manufacturing patent notice challenge: a comparative examination
Source: J Law Biosci. 2026 Aug 14;13(2):lsaf025. doi: 10.1093/jlb/lsaf025 (PMC13474269; doi:10.1093/jlb/lsaf025)
Supplement: Supplementary file 1 [file supplementary_materials_lsaf025.zip › Appendix C.docx]

Table S5. Account of BPCIA filed complaints by Regeneron against five biosimilar applicants

| **Complaint filing date** | **Defendant** | **Accused biosimilar** | **Notification date of FDA acceptance to review the aBLA application** | **Step1-Patent dance** | **Step2** | **Step3** | **Step 4** | **Marketing status** | **Launched** | **Reason** |
| --- | --- | --- | --- | --- | --- | --- | --- | --- | --- | --- |
| Aug. 2, 2022 | Mylan / Biocon (Viatris) | YESAFILI (aflibercept-jbvf) | 28-Dec-21 | Mylan informed regeneron about its aBLA M710 acceptance and provided the application | Regeneron supplied a patent list on the original and another on the supplement | Mylan alleged that any of Regeneron patents is either invalid or unenforceable | Regeneron brought an additional list of patents that were issued following Regeneron’s service of its original patent list in Step 2. The complaint for an infringement over 24 patents and on the Mylan’s claim that it will launch ahead of the end of Eylea’s exclusivity period | FDA approved on May 20, 2024 (BLA 761274) | no | Permanent injunction |
| Nov 8, 2023 | Celltrion | CT-P42 (aflibercept) | 1-Sep-23 | On September 13, 2023, Celltrion served a copy of its Biologics License Application for CT-P42 (“Celltrion aBLA”) and also its Notice of Commercial Marketing immediately after FDA approval. | On November 7, 2023, Regeneron served on Celltrion a list of patents believed a claim of patent infringement could reasonably be asserted (“3A list”) | Regeneron claimed that in 2022 and 2023 shipments of “AFLIBERCEPT  (INHIBITOR (GROWTH FACTOR))” was imported into the United States and it believes they were intended for commercial use. | Complaint filed for a judgment of infringement and declaratory judgment of infringement of patents. | Not approved | no | Preliminary injunction |
| Nov 22, 2023 | Samsung Bioepis Co., Ltd. | OPUVIZ (aflibercept-yszy) | February 15, 2024 | Text in the complaint was redacted and so we could not confirm whether Samsung Bioepis participated in the patent dance. But from the FDA review file dated February 15, 2024 , we learned that Samsung Bioepis submitted BLA 761350 on February 17, 2023 seeking FDA approval for SB15 (Opuviz) as an interchangeable biosimilar to Eylea (aflibercept). The product meets the requirements for licensure as interchangeable with the corresponding presentation of Eylea (aflibercept) injection, 2 mg/0.05 mL for intravitreal use and so FDA issued a conditional acceptance to review the application but because approval of this BLA is precluded by an unexpired pediatric and reference product exclusivity periods, FDA could not approve this product. Regeneron filed the complaint to obtain relief before Bioepis launches its Eylea biosimilar. The firm alleged that Samsung Bioepis “infringed a common set of thirteen U.S. patents covering EYLEA, by submitting aBLAs and seeking to market their follow-on biologic products. Regeneron also claimed that between August 29, 2021 and March 28, 2023, Bioepis imported or directed one or more of its subsidiaries, affiliates, or agents to import 17 shipments of SB15 into the United States. The shipments contained a product labelled:“AFLIBERCEPT (INHIBITOR (GROWTH FACTOR)).” | | | | Approved on 05/20/2024 (BLA 761350) | no | Preliminary injunction |
| Nov 29, 2023 | Formycon | AHZANTIVE (FYB203) (aflibercept-mrbb) | August 29, 2023 | Similar to Samsung and Celltrion’s cases. Regeneron claimed that Formycon's submission of its aBLA before the end of exclusivity period constitutes an act of patent infringement and filed a complaint to obtain relief ahead of FDA approval. | | | | Approved on 06/28/2024 (BLA 761378) |  | Preliminary injunction |
| Jan 10, 2024 | Amgen | ABP 938 (aflibercept) | October 31, 2023 | Amgen provided its aBLA but not its manufacturing process | Regeneron provided the list of asserted patents | Amgen replied that patents of Regeneron’s 3(A) List are invalid or will not be infringed. | After a series of interactions, Amgen did not respond within the required 30 days, so Regeneron filed a complaint seeking action in a judgement of infringement on US patents in suit | BLA 761298/split into # applications | pending |  |
| August 2024 | Amgen | PAVBLU (aflibercept-ayyh) |  |  |  | Regeneron and Amgen agreed to have a hearing on injunction proceedings against Amgen until August 2024. | Between September and October 2024, the district court and Federal Circuit denied Regeneron’s request for an injunction. The judge's decision explaining his denial was issued under seal | Approved on 08/23/2024 (BLA 761298/Original 1) | YES | Nov 5, 2024 and litigation pending? |
| Aug 26,2024 | Sandoz | ENZEEVU (aflibercept-abzv) | August 12, 2024 | Sandoz did not initiate the patent dance. On August 12, 2024, Sandoz announced via a press release that FDA granted approval for ENZEEVU in both vial and pre-filled syringe presentations | Regeneron sent two letters to Sandoz inquiring about its aBLA application | Sandoz notified Regeneron that the application was approved | Regeneron filed a complaint because Sandoz  did not timely provide any notice to Regeneron nor participated in the BPCIA framework | Approved on 08/09/2024 (BLA 761382/Original 1) | no | Litigation pending |

Table S6. List of Regeneron’s asserted patents against each of the biosimilar applicants and which matched in our dataset. Alternate shading of rows denotes the represented families. Within each family, patent members are arranged based on their earliest filing date and their patent type. Biosimilar applicants’ columns are colour coded to highlight the overlapping asserted patents.

|  |  | **Biosimilar applicant (filing date of BPCIA complaint by Regeneron)** | | | | | | |  |  |
| --- | --- | --- | --- | --- | --- | --- | --- | --- | --- | --- |
| **Regeneron's asserted patents that matched in our dataset** | **Patent type** | **Mylan (Aug. 2, 2022)** | **Celltrion (Nov. 8, 2023)** | **Bioepis 1 (Nov. 22, 2023)** | **Bioepis-2 (Dec. 27, 2023)** | **Formycon (Nov. 29, 2023)** | **Amgen (Jan 10, 2024)** | **Sandoz (Aug. 26, 2024)** | **Title** | **Filing date** |
| US 10406226 B2 | Continuation | X |  |  |  |  |  |  | Method of manufacturing VEGF antagonist fusion proteins | 8/31/17 |
| US 11053280 B2 | original | X | X | X | X | X |  | X | Anti-VEGF protein compositions and methods for producing the same | 8/18/20 |
| US 11104715 B2 | original | X | X | X | X | X | X | X | Methods for producing aflibercept in chemically defined media having reduced aflibercept variants | 8/18/20 |
| US 11186625 B2 | original | X |  |  |  |  |  |  | Anti-VEGF protein compositions and methods for producing the same | 8/18/20 |
| US 11174283 B2 | Continuation | X | X | X | X | X |  | X | Anti-VEGF protein compositions and methods for producing the same | 3/18/21 |
| US 11535663 B2 | Continuation |  | X | X | X | X | X | X | Methods for producing aflibercept in chemically defined media having reduced aflibercept variants | 7/22/21 |
| US 11306135 B2 | Continuation | X | X | X | X | X | X |  | Anti-VEGF protein compositions and methods for producing the same | 8/30/21 |
| US 11299532 B2 | 2nd continuation* | X | X | X | X | X |  | X | Anti-VEGF protein compositions and methods for producing the same | 9/29/21 |
| US 11459373 B2 | 3rd continuation |  |  |  |  |  |  | X | Anti-VEGF protein compositions and methods for producing the same | 10/13/21 |
| US 11472861 B2 | 2nd continuation |  | X | X | X | X | X | X | Methods for producing aflibercept in chemically defined media having reduced aflibercept variants | 12/21/21 |
| US 11485770 B2 | Division |  | X | X | X | X |  |  | Anti-VEGF protein compositions and methods for producing the same | 1/13/22 |
| US 11459374 B2 | 4th continuation |  | X |  | X |  | X | X | Anti-VEGF protein compositions and methods for producing the same | 2/8/22 |
| US 11505593 B2 | continuation |  | X |  | X |  | X |  | Anti-VEGF protein compositions and methods for producing the same | 2/8/22 |
| US 11548932 B2 | 3rd continuation |  | X | X | X | X | X | X | Anti-VEGF protein compositions and methods for producing the same | 6/15/22 |
| US 11542317 B1 | 2nd continuation |  | X | X | X | X | X |  | Anti-VEGF protein compositions and methods for producing the same | 6/16/22 |
| US 11753459 B2 | Continuation |  | X | X | X | X | X |  | Anti-VEGF protein compositions and methods for producing the same | 7/6/22 |
| US 11732025 B2 | 4th continuation |  |  |  |  |  |  | X | Anti-VEGF protein compositions and methods for producing the same | 11/25/22 |
| US 11332771 B2 | Continuation |  |  |  |  |  |  | X | Serum-free cell culture medium | 1/14/20 |
| US 11970724 B2 | 2nd continuation |  |  |  |  |  |  | X | Serum-free cell culture medium | 7/8/21 |
| US 10669594 B2 | original | X | X | X | X | X | X | X | Compositions and methods for detecting a biological contaminant | 3/25/16 |
| US 11549154 B2 | Division |  |  |  |  | X |  |  | Compositions and methods for detecting a biological contaminant | 4/22/20 |
| US 11680930 B2 | 2nd continuation |  |  |  |  | X | X |  | Methods and systems for chromatography data analysis | 4/18/22 |
| US 10918754 B2 | Bypass continuation |  |  | X | X |  | X | X | Sterilisation method | 3/6/18 |
| US 10905786 B2 | Continuation |  |  | X | X |  | X | X | Sterilisation method | 12/4/19 |
| US 7070959 B1** | Bypass continuation | X | X | X |  | X |  |  | Modified chimeric polypeptides with improved pharmacokinetic properties | 12/6/01 |
| US 11525833 B2 | original |  | X |  |  |  |  |  | Hydrophobic interaction chromatography-coupled native mass spectrometry for antibody analysis | 9/27/20 |
| US 9816110 B2 | original | X | X | X | X | X | X | X | CHO integration sites and uses thereof | 10/21/15 |
| US 11788102 B2 | 2nd continuation |  | X | X | X | X | X | X | CHO integration sites and uses thereof | 9/19/19 |
| US 11268109 B2 | 3rd continuation |  |  |  |  | X |  | X | CHO integration sites and uses thereof | 2/12/21 |
| US 11555176 B2 | 2nd continuation |  | X | X | X |  | X | X | Cell culture medium for eukaryotic cells | 2/22/22 |
| US 7771997 B2 | original |  |  |  |  | X |  |  | Enhanced expression and stability regions | 6/4/08 |
| US 9222106 B2*** | Division of a continuation | X | X | X | X | X | X | X | Enhanced expression and stability regions | 1/29/13 |
| US 9562238 B2 | continuation of a division |  |  | X | X | X |  |  | Enhanced expression and stability regions | 12/14/15 |
| US 9932605 B2 | 2nd continuation of a division |  |  |  |  | X |  |  | Enhanced expression and stability regions | 12/20/16 |
| US 10415055 B2 | 3rd continuation of a division | X | X | X | X | X | X | X | Enhanced expression and stability regions | 3/9/18 |
| US 11312936 B2 | original |  | X | X | X | X |  | X | Taurine supplemented cell culture medium and methods of use | 8/3/16 |
| US 10927342 B2 | Continuation | X | X | X | X | X |  | X | Taurine supplemented cell culture medium and methods of use | 1/14/20 |

* 2nd continuation means it is a continuation of another continuation..

** This patent was issued on July 4, 2006 but it was subject to a terminal disclaimer with anticipated termination date of May 23, 2020. Also this patent has no equivalent document in Europe.

*** On information and believe, Regeneron asserted claim 20 of this patent which is a manufacturing platform general claim: *“A method for making a protein of interest, comprising:
(a) providing a recombinant host cell that comprises an expression-enhancing sequence selected from a sequence of SEQ ID NO:1-4 and at least one recombinase recognition site within or immediately adjacent to the expression-enhancing sequence; (b) introducing into the host cell a nucleic acid construct within the expression-enhancing sequence comprising a GOI that encodes for a protein of interest;
(c) culturing the host cell of under conditions that allow expression of the GOI; and,
(d) recovering the protein of interest.”*
