# Supplementary material for: The biologics manufacturing patent notice challenge: a comparative examination
Source: J Law Biosci. 2026 Aug 14;13(2):lsaf025. doi: 10.1093/jlb/lsaf025 (PMC13474269; doi:10.1093/jlb/lsaf025)
Supplement: Supplementary file 1 [file supplementary_materials_lsaf025.zip › Revised Appendix A_20250919.docx]

Appendix A

Table S1. Family size and filing timespan of the selected 88 families. For each selected family within a firm, we computed the total family size (all patent applications and grants available internationally on November 5, 2024 in the Lens platform), the U.S.-only family size and filing timespan (number of U.S. grants and applications family members from 1st filing to last filing on November 5, 2024), and the number of U.S.patent members analyzed. Entries shaded in blue show U.S. filings with no foreign equivalents.

Table S2. Analysis of first and 2nd filings of each of the 88 selected families representing all the leading nine originator biologics firms. For each family, we list the numbers of provisional applications used to start the family.

Table S3. Regeneron’s family 49 entitled “Modified chimeric polypeptides with improved pharmacokinetic properties” began as a US provisional application No. 60/138,133, filed on Jun. 8, 1999, then PCT application filed on May 23, 2000, PCT/US00/14142 § 371 (c)(1), (2), (4) filed Dec 6, 2001 (PCT Pub No. was WO00/75319 published on December 14, 2000). These were followed by bypass divisionals/continuations until 2017. The actual patent number of each of the 13 issued family members, the patent type, filing date, and corresponding independent claims are depicted below.

| Patent | Filing date | Independent claims | Patent type | Continuation type |
| --- | --- | --- | --- | --- |
| [US 7070959 B1](https://www.lens.org/lens/patent/129-358-003-332-698/frontpage?l=en) | 12/6/01 | *"1. An isolated nucleic acid molecule encoding a fusion protein capable of binding vascular endothelial growth factor (VEGF), consisting of (a) a (VEGF) receptor component having immunoglobulin-like (Ig) domains consisting of an Ig domain 2 of a first VEGF receptor human Flt1 and Ig domain 3 of a second VEGF receptor human Flk1; and (b) a multimerizing component."*  *"7. An expression vector comprising a nucleic acid molecule encoding a fusion protein capable of binding vascular endothellal growth factor (VEGF), wherein the fusion protein consists of immunoglobulin-like (Ig) domain 2 of VEGF receptor human Flt1, Ig domain 3 of VEGF receptor human Flk1, and a multimerizing component."*  *"8. A host-vector system for the production of a fusion polypeptide comprising an expression vector encoding a fusion protein capable of binding vascular endothelial growth factor (VEGF), wherein the fusion protein consists of immunoglobulin-like (Ig) domain 2 of VEGF receptor human Flt1, Ig domain 3 of VEGF receptor human Flk1, and a multimerizing component, In a suitable isolated host cell."*  *"12. An isolated nucleic acid molecule encoding a fusion protein capable of binding vascular endothelial growth factor (VEGF), consisting of (a) a VEGF receptor component having immunoglobulin-like (Ig) domains consisting of an Ig domain 2 of a first VEGF receptor human Flt1 and Ig domain 3 of a second VEGF receptor human Flt-4: and (b) a multimerizing component."*  *"14. An isolated nucleic acid molecule consisting of a nucleotide sequence encoding immunoglobulin-like (Ig) domain 2 of a first vascular endothelial growth factor (VEGF) receptor upstream of a nucleotide sequence encoding Ig domain 3 of a second VEGF receptor and a nucleotide sequence encoding a multimerizing component, wherein the nucleic acid sequence is SEQ ID NO:15."* | Original | Bypass application under 35 U.S.C. § 371 (c)(1), (2), (4) |
| [US 7374757 B2](https://www.lens.org/lens/patent/171-386-071-328-472/frontpage?l=en) | 12/17/04 | *"1. A fusion protein, consisting of (a) a vascular endothelial growth factor (VEGF) receptor component having immunoglobulin-like (Ig) domains consisting of an Ig domain 2 of a first VEGF receptor human Flt1 and Ig domain 3 of a second VEGF receptor human Flk1; and (b) a multimerizing component, wherein the fusion protein binds VEGF."*  *"6. A fusion protein encoded by a nucleic acid sequence selected from: (a) SEQ ID NO:15; and (b) nucleic acid sequences which, as a result of the degeneracy of the genetic code, differ from the nucleic acid sequence of SEQ ID NO:15."*  *"7. A fusion protein consisting of immunoglobulin-like (Ig) domain 2 of a first vascular endothelial growth factor (VEGF) receptor upstream of Ig domain 3 of a second VEGF receptor and a multimerizing component, wherein the fusion protein comprises the amino acid sequence SEQ ID NO:16."* | Bypass division | Division of application No. 10/009,852, filed as application No. PCT/US00/14142 on May 23, 2000, now Pat. No. 7,070,959 |
| [US 7374758 B2](https://www.lens.org/lens/patent/085-823-872-746-338/frontpage?l=en) | 12/17/04 | *"1. A method of inhibiting vascular endothelial growth factor (VEGF) activity in a mammal, comprising: administering a pharmaceutical composition to the mammal, wherein the pharmaceutical composition comprises (a) a VEGF antagonist, and (b) a pharmaceutically acceptable carrier wherein the VEGF antagonist comprises a dimeric fusion polypeptide comprising two fusion polypeptides, each fusion polypeptide comprising: (i) a VEGF receptor component consisting of an immunoglobulin-like (Ig) domain 2 of a first VEGF receptor human Flt1 and Ig domain 3 of a second VEGF receptor human Flk1 or human Flt4; and (ii) a multimerizing component, wherein VEGF activity is inhibited."*  *"3. A method of inhibiting tumor growth in a mammal, comprising: administering a pharmaceutical composition to the mammal, wherein the pharmaceutical composition comprises (a) a VEGF antagonist, and (b) a pharmaceutically acceptable carrier wherein the VEGF antagonist comprises a dimeric fusion polypeptide comprising two fusion polypeptides, each fusion polypeptide comprising: (i) a VEGF receptor component consisting of an immunoglobulin-like (Ig) domain 2 of a first VEGF receptor human Flt1 and Ig domain 3 of a second VEGF receptor human Flk1 or human Flt4; and (ii) a multimerizing component, wherein tumor growth is inhibited."* | Bypass division | Division of application No. 10/009,852, filed as application No. PCT/US00/14142 on May 23, 2000, now Pat. No. 7,070,959 |
| [US 7524499 B2](https://www.lens.org/lens/patent/128-634-823-596-957/frontpage?l=en) | 4/14/08 | *"1. A fusion protein, consisting of a vascular endothelial growth factor (VEGF) receptor component having immunoglobulin-like (Ig) domains consisting of an Ig domain 2 of a first VEGF receptor human Flt1 and an Ig domain 3 of a second VEGF receptor human Flt-4; and a multimerizing component."* | 2nd division | Division of application No. 11/016,097, filed on Dec. 17, 2004, now Pat. No. 7,374,757, which is a division of application No. 10/009,852, filed as application No. PCT/US00/14142 on May 23, 2000, now Pat. No. 7,070,959 |
| [US 7704500 B2](https://www.lens.org/lens/patent/150-432-160-954-208/frontpage?l=en) | 4/14/08 | *"1. A method of attenuating or reducing formation of ascites in a mammal, comprising administering to the mammal an effective amount of a fusion protein vascular endothelial growth factor (VEGF) antagonist, wherein the fusion protein consists of immunoglobulin (Ig)-like domain 2 of a first VEGF receptor human Flt1 and Ig-like domain 3 of a second VEGF receptor human Flk1, and a multimerizing component."*  *"6. A method of inhibiting the production of ascites associated with ovarian carcinoma, comprising administering to a human an effective amount of a fusion protein vascular endothelial growth factor (VEGF) antagonist, wherein the fusion protein consists of immunoglobulin (Ig)-like domain 2 of a first VEGF receptor human Flt1 and Ig-like domain 3 of a second VEGF receptor human Flk1, and a multimerizing component."* | 2nd division | Division of application No. 11/016,503, filed on Dec. 17, 2004, now Pat. No. 7,374,758, which is a division of application No. 10/009,852, filed as application No. PCT/US00/14142 on May 23, 2000, now Pat. No. 7,070,959 |
| [US 8029791 B2](https://www.lens.org/lens/patent/037-405-941-158-052/frontpage?l=en) | 4/14/08 | *"1. A dimeric vascular endothelial growth factor (VEGF) antagonist, comprising two fusion proteins, each fusion protein consisting of (a) a VEGF receptor component having immunoglobulin-like (Ig) domains consisting of an Ig domain 2 of a first VEGF receptor human Flt1 and Ig domain 3 of a second VEGF receptor human Flk1; and (b) a multimerizing component."*  *"5. A dimeric vascular endothelial growth factor (VEGF) antagonist, comprising two fusion proteins, each fusion protein consisting of (a) a VEGF receptor component having immunoglobulin-like (Ig) domains consisting of an Ig domain 2 of a first VEGF receptor human Flt1 and Ig domain 3 of a second VEGF receptor human Flk1; and (b) an Fc domain of IgG."*  *"6. A composition comprising a VEGF antagonist and a carrier, wherein the VEGF antagonist comprises two fusion proteins, each fusion protein consisting of (a) a VEGF receptor component having immunoglobulin-like (Ig) domains consisting of an Ig domain 2 of a first VEGF receptor human Flt1 and Ig domain 3 of a second VEGF receptor human Flk1; and (b) a multimerizing component."* | Continuation | Continuation of application No. 11/016,097, filed on Dec. 17, 2004, now Pat. No. 7,374,757, which is a division of application No. 10/009,852, filed as application No. PCT/US00/14142 on May 23, 2000, now Pat. No. 7,070,959 |
| [US 7964377 B2](https://www.lens.org/lens/patent/156-414-057-303-342/frontpage?l=en) | 12/15/08 | *"1. An isolated nucleic acid molecule encoding a fusion protein capable of binding vascular endothelial growth factor (VEGF) consisting of: (a) a VEGF receptor component having immunoglobulin-like (Ig) domain consisting of an Ig domain 2 of a first VEGF receptor human Flt1 and an Ig domain 3 of a second VEGF receptor human Flt4; and (b) a multimerizing component, wherein the nucleic acid molecule is the nucleic acid sequence of SEQ ID NO:13, or a nucleotide sequence which, as a result of the degeneracy of the genetic code, differs from SEQ ID NO:13."* | Continuation | Continuation of application No. 12/102,648, filed on Apr. 14, 2008, now Pat. No. 7,524,499, which is a division of application No. 11/016,097, filed on Dec. 17, 2004, now Pat. No. 7,374,757, which is a division of application No. 10/009,852, filed as application No. PCT/US00/14142 on May 23, 2000, now Pat. No. 7,070,959 |
| [US 8084234 B2](https://www.lens.org/lens/patent/145-985-199-674-624/frontpage?l=en) | 3/1/10 | *"1. An isolated nucleic acid molecule encoding a fusion protein capable of binding vascular endothelial growth factor (VEGF), consisting of (a) a (VEGF) receptor component having immunoglobulin-like (Ig) domains consisting of an Ig domain 2 of a first VEGF receptor human Flt1 and Ig domain 3 of a second VEGF receptor human Flt4; and (b) a multimerizing component."*  *"7. An expression vector comprising a nucleic acid molecule encoding a fusion protein capable of binding vascular endothelial growth factor (VEGF), wherein the fusion protein consists of immunoglobulin-like (Ig) domain 2 of VEGF receptor human Flt1, Ig domain 3 of VEGF receptor human Flt4, and a multimerizing component."*  *"8. An isolated host cell for the production of a fusion polypeptide, wherein the host cell comprises an expression vector comprising a nucleic acid molecule encoding a fusion protein capable of binding vascular endothelial growth factor (VEGF), wherein the fusion protein consists of immunoglobulin-like (Ig) domain 2 of VEGF receptor human Flt1, Ig domain 3 of VEGF receptor human Flt4, and a multimerizing component."*  *"12. An isolated nucleic acid molecule consisting of a nucleotide sequence encoding immunoglobulin-like (Ig) domain 2 of a first vascular endothelial growth factor (VEGF) receptor upstream of a nucleotide sequence encoding Ig domain 3 of a second VEGF receptor and a nucleotide sequence encoding a multimerizing component, wherein the nucleic acid sequence is SEQ ID NO:13."* | Continuation | Continuation of application No.12/102,681, filed on Apr. 14, 2008, now Pat. No. 7,704,500, which is a division of application No. 11/016,097, filed on Dec. 17, 2004, now Pat. No. 7,374,757, which is a division of application No. 10/009,852, filed as application No. PCT/US00/14142 on May 23, 2000, now Pat. No. 7,070,959 |
| [US 8343737 B2](https://www.lens.org/lens/patent/084-992-084-833-167/frontpage?l=en) | 5/13/11 | *"1. A cell culture composition comprising cells suspended in culture medium, wherein the cells contain an expression vector comprising a nucleic acid molecule that encodes the amino acid sequence of Flt1D2.Flk1D3.FcΔC1(a) (SEQ ID NO:12) or VEGFR1R2-FcΔC1(a) (SEQ ID NO:16)."* | 2nd continuation | Continuation of application No. 12/334,927, filed on Dec. 15, 2008, now Pat. No. 7,964,377, which is a continuation of application 12/102,648, filed on Apr. 14, 2008, now Pat. No. 7,524,499, which is a division of application No. 11/016,097, filed on Dec. 17, 2004, now Pat. No. 7,374,757, which is a division of application No. 10/009,852, filed as application No. PCT/US00/14142 on May 23, 2000, now Pat. No. 7,070,959 |
| [US 8647842 B2](https://www.lens.org/lens/patent/037-570-812-116-162/frontpage?l=en) | 4/5/12 | *"1. A method for producing a fusion protein capable of binding VEGF, the method comprising: (a) growing recombinant cells in suspension culture, wherein the recombinant cells contain an expression vector comprising a nucleic acid molecule that encodes a fusion protein selected from the group consisting of Flt1D2.Flk1D3.FcΔC1(a) (SEQ ID NO:12) and VEGFR1R2-FcΔC1(a) (SEQ ID NO:16); and (b) isolating the fusion protein from the suspension culture."*  *"13. A method for producing a fusion protein capable of binding VEGF, the method comprising: (a) incubating recombinant cells in T-225 cm 2 flasks in culture medium at 37° C. and 5% CO 2 until the cells approach confluence; (b) transferring the cells from the T-225 cm 2 flasks to roller bottles and incubating the cells at 37° C. and 5% CO 2 until the cells reach confluence; (c) transferring the cells from the roller bottles to a 5 L bioreactor and growing the cells in 3.5 L of suspension culture at 37° C. until the cell density reaches 4×10 6 cells/mL; (d) transferring the cells from the 5 L bioreactor to a 40 L bioreactor and growing the cells in suspension culture at 34° C.; and (e) isolating the fusion protein from the suspension culture from the 40 L bioreactor by affinity chromatography, followed by size-exclusion chromatography; wherein the recombinant cells contain an expression vector comprising a nucleic acid molecule that encodes a fusion protein selected from the group consisting of Flt1D2.Flk1D3.FcΔC1(a) (SEQ ID NO:12) and VEGFR1R2-FcΔC1(a) (SEQ ID NO:16)."* | 3rd continuation | Continuation of application No. 12/885,185, filed on Sep. 17, 2010, now abandoned, which is a continuation of application No. 12/715,128, filed on Mar. 1, 2010, now Pat. No. 8,084,234, which is a continuation of application No.12/102,681, filed on Apr. 14, 2008, now Pat. No. 7,704,500, which is a division of application No. 11/016,097, filed on Dec. 17, 2004, now Pat. No. 7,374,757, which is a division of application No. 10/009,852, filed as application No. PCT/US00/14142 on May 23, 2000, now Pat. No. 7,070,959 |
| [US 9139644 B2](https://www.lens.org/lens/patent/059-490-080-121-158/frontpage?l=en) | 12/18/13 | *"1. A chimeric polypeptide capable of antagonizing VEGF activity consisting of a modified extracellular ligand binding domain of a VEGF receptor fused to a multimerizing component, wherein the modified extracellular ligand binding domain consists of an immunoglobulin-like (Ig) domain of human Flt1 and an Ig domain of human Flk1, wherein the Ig domain of human Flt1 is Ig domain 2, and wherein the Ig domain of human Flk1 is Ig domain 3, wherein the chimeric polypeptide exhibits improved pharmacokinetic properties as compared to a chimeric polypeptide consisting of only Ig domains of Flt1."* | 4th continuation | Continuation of application No. 13/439,889, filed on Apr. 5, 2012, now Pat. No. 8,647,842, which is a continuation of application No. 12/885,185, filed on Sep. 17, 2010, now abandoned, which is a continuation of application No. 12/715,128, filed on Mar. 1, 2010, now Pat. No. 8,084,234, which is a  continuation of application No.12/102,681, filed on Apr. 14, 2008, now Pat. No. 7,704,500, which is a division of application No. 11/016,097, filed on Dec. 17, 2004, now Pat. No. 7,374,757, which is a division of application No. 10/009,852, filed as application No. PCT/US00/14142 on May 23, 2000, now Pat. No. 7,070,959 |
| [US 9708386 B2](https://www.lens.org/lens/patent/037-096-018-601-245/frontpage?l=en) | 8/11/15 | *"1. A chimeric polypeptide capable of antagonizing VEGF activity consisting of a modified extracellular ligand binding domain of a VEGF receptor fused to a multimerizing component, wherein the modified extracellular ligand binding domain consists essentially of an immunoglobulin-like (Ig) domain of human Flt1 and an Ig domain of human Flt1wherein the Ig domain of human Flt1is Ig domain 2, and wherein the Ig domain of human Flk1is Ig domain 3 , wherein the chimeric polypeptide exhibits improved pharmacokinetic properties as compared to a chimeric polypeptide consisting of only Ig domains of Flt1."* | 5th continuation | Continuation of application No. 14/109,999, filed on Dec. 18, 2013, now Pat. No. 9,139,644, which is a continuation of application No. 13/439,889, filed on Apr. 5, 2012, now Pat. No. 8,647,842, which is a continuation of application No. 12/885,185, filed on Sep. 17, 2010, now abandoned, which is a continuation of application No. 12/715,128, filed on Mar. 1, 2010, now Pat. No. 8,084,234, which is a  continuation of application No.12/102,681, filed on Apr. 14, 2008, now Pat. No. 7,704,500, which is a division of application No. 11/016,097, filed on Dec. 17, 2004, now Pat. No. 7,374,757, which is a division of application No. 10/009,852, filed as application No. PCT/US00/14142 on May 23, 2000, now Pat. No. 7,070,959 |
| [US 10392430 B2](https://www.lens.org/lens/patent/143-210-879-581-433/frontpage?l=en) | 6/16/17 | *"1. A method for making a nucleic acid molecule encoding a chimeric VEGF-binding fusion protein with improved pharmacokinetic properties, the method comprising: modifying a nucleic acid molecule encoding immunoglobulin (“Ig”) domains 1, 2 and 3 of Flt1 fused to an Fc domain (“Flt1(1-3)-Fc”), wherein said modifying comprises: (a) deleting the nucleotide sequence encoding the first Ig domain of Flt1; and (b) replacing the nucleotide sequence encoding the third Ig domain of Flt1 with the nucleotide sequence encoding the third Ig domain of Flk1 or Flt4; thereby making a nucleic acid molecule encoding a chimeric VEGF-binding fusion protein with reduced binding to extracellular matrix (ECM) and improved pharmacokinetics (PK) as compared to Flt1(1-3)-Fc."* | 6th continuation | Continuation of application No. 14/822,929, filed on Aug. 11, 2015, now Pat. No. 9,708,386 which is a continuation of application No. 14/109,999, filed on Dec. 18, 2013, now Pat. No. 9,139,644, which is a continuation of application No. 13/439,889, filed on Apr. 5, 2012, now Pat. No. 8,647,842, which is a continuation of application No. 12/885,185, filed on Sep. 17, 2010, now abandoned, which is a continuation of application No. 12/715,128, filed on Mar. 1, 2010, now Pat. No. 8,084,234, which is a  continuation of application No.12/102,681, filed on Apr. 14, 2008, now Pat. No. 7,704,500, which is a division of application No. 11/016,097, filed on Dec. 17, 2004, now Pat. No. 7,374,757, which is a division of application No. 10/009,852, filed as application No. PCT/US00/14142 on May 23, 2000, now Pat. No. 7,070,959 |

Table S4. Regeneron’s family 18 has 31 issued patents under two titles: “*Methods for producing aflibercept in chemically defined media having reduced aflibercept variants*” (patents US 11104715 B2, US 11535663 B2, and US 11472861 B2) and “*Anti-VEGF protein compositions and methods for producing the same*” for the remaining patents. The family began following two US provisional applications No. 62/944,635, filed on Dec. 6, 2019, and No. 63/065,012, filed on Aug. 13, 2020. These led to six original patents followed by a series of divisions/continuations until June 2023. The granted patents, which were asserted against Eylea biosimilar applications, are marked in red and due to space limitation, we list mostly independent claim 1 for reference here, but we provide a link to the actual patent document in the Lens platform for reference.

| **Family Members** | **Filing Date** | **Relevant Claim** | **Patent type** | **Continuation type** |
| --- | --- | --- | --- | --- |
| [US 11053280 B2](https://lens.org/124-543-785-137-944) | 8/18/20 | *"1. A method of producing aflibercept, comprising: (a) producing a clarified harvest of cells cultured in a chemically defined medium (CDM); (b) binding aflibercept from said clarified harvest to a Protein A resin, wherein said aflibercept includes variants that have at least one oxidized amino acid residue selected from the group consisting of methionine, tryptophan, histidine, phenylalanine, tyrosine and a combination thereof; (c) eluting said aflibercept of step (b) forming an affinity eluate, wherein said eluate has a first color; (d) subjecting said eluate comprising aflibercept to an anion exchange chromatography (AEX) column; and (e) collecting a flowthrough fraction, wherein said flowthrough fraction has a second color, and wherein said first color of said affinity eluate is a more intense yellow brown color than said second color of said flowthrough fraction when said affinity eluate and flowthrough fraction protein concentrations are normalized."* | Original | US 2021/0171570 A1 Jun. 10, 2021 Provisional application No. 63/065,012, filed on Aug. 13, 2020.  Provisional application No. 62/944,635, filed on Dec. 6, 2019 |
| [US 11098112 B2](https://lens.org/120-479-826-208-00X) | 8/18/20 | *"1. A method of producing aflibercept, comprising: (a) producing a clarified harvest of cells cultured in a chemically defined medium (CDM); (b) binding aflibercept from said clarified harvest using an affinity chromatography column comprising a polypeptide capable of binding to or interacting with said aflibercept, wherein said polypeptide is an antibody, a fusion protein, a ScFv or a fragment thereof and wherein said polypeptide comprises an isolated amino acid sequence selected from a SEQ ID NO.: 72, SEQ ID NO.: 73, SEQ ID NO.: 74, SEQ ID NO.: 75, SEQ ID NO.: 76, SEQ ID NO.: 77, SEQ ID NO.: 78, SEQ ID NO.: 79 and SEQ ID NO.: 80; (c) eluting said aflibercept of step (b) forming an affinity eluate; optionally, (d) subjecting said eluted aflibercept of (c) to a second chromatography capture step; and (e) collecting a flowthrough fraction, wherein said flowthrough fraction has aflibercept."* | Original | US 2021/0214430 A1 Jul. 15, 2021  Provisional application No. 63/065,012, filed on Aug. 13, 2020.  Provisional application No. 62/944,635, filed on Dec. 6, 2019. |
| [US 11098311 B2](https://lens.org/167-741-534-093-788) | 8/18/20 | *"1. An isolated, synthetic, or recombinant polypeptide having IdeS protease activity comprising the amino acid sequence selected from the group consisting of SEQ ID NO.: 2, SEQ ID NO.: 3, SEQ ID NO.: 4, SEQ ID NO.: 5, SEQ ID NO.: 6, SEQ ID NO.: 7, SEQ ID NO.: 8, SEQ ID NO.: 9, SEQ ID NO.: 10, SEQ ID NO.: 11, SEQ ID NO.: 12, SEQ ID NO.: 13, SEQ ID NO.: 14, SEQ ID NO.: 15 and SEQ ID NO.: 16."*    *"10. An isolated nucleic acid molecule comprising a nucleotide sequence encoding a polypeptide having IdeS protease activity comprising the amino acid sequence selected from the group consisting of SEQ ID NO.: 2, SEQ ID NO.: 3, SEQ ID NO.: 4, SEQ ID NO.: 5, SEQ ID NO.: 6, SEQ ID NO.: 7, SEQ ID NO.: 8, SEQ ID NO.: 9, SEQ ID NO.: 10, SEQ ID NO.: 11, SEQ ID NO.: 12, SEQ ID NO.: 13, SEQ ID NO.: 14, SEQ ID NO.: 15 and SEQ ID NO.: 16."*  *"11. An isolated synthetic, or recombinant polypeptide having IdeS protease activity comprising the full length amino acid sequence set forth in SEQ ID NO.: 1 with asparagine residues at one or more positions of 87, 130, 182 or 274 mutated to an amino acid other than asparagine, wherein said amino acid is aspartic acid, leucine, or arginine."* | Original | US 2021/0214733 A1 Jul. 15, 2021. Provisional application No. 63/065,012, filed on Aug. 13, 2020. Provisional application No. 62/944,635, filed on Dec. 6, 2019 |
| [US 11104715 B2](https://lens.org/043-253-009-663-851) | 8/18/20 | *"1. A method of producing aflibercept harvested from a host cell cultured in a chemically defined medium (CDM), comprising: (a) providing a host cell genetically engineered to express aflibercept; (b) culturing said host cell in said CDM under conditions suitable in which said host cell expresses said aflibercept wherein the cumulative concentration of nickel in said CDM is less than or equal to 0.4 μM or about 0.4 μM and one or more of the following: i. the cumulative concentration of iron in said CDM is less than or equal to 55.0 μM; ii. the cumulative concentration of copper in said CDM is less than or equal to 0.8 μM; iii. the cumulative concentration of zinc in said CDM is less than or equal to 56.0 μM; iv. the cumulative concentration of cysteine in said CDM is less than or equal to 10.0 mM, and v. said CDM includes anti-oxidants where the cumulative concentration of an antioxidant is about 0.001 mM to about 10.0 mM for any single anti-oxidant; and (c) harvesting aflibercept produced by said host cell."* | Original | US 2021/0206832 A1 Jul. 8, 2021  Provisional application No. 63/065,012, filed on Aug. 13, 2020.  Provisional application No. 62/944,635, filed on Dec. 6, 2019. |
| [US 11180540 B2](https://lens.org/045-010-737-504-548) | 8/18/20 | *"1. A method of producing aflibercept MiniTrap from a clarified harvest of a cell cultured in a chemically defined medium (CDM) and expressing aflibercept, comprising: (a) binding aflibercept from said clarified harvest to a first capture chromatography; (b) eluting said aflibercept of step (a) and subjecting said aflibercept to enzymatic cleavage to remove its Fc domain thereby forming MiniTrap; (c) subjecting (b) to a second capture chromatography, wherein said second capture chromatography step is subjected to one or more washes, and wherein a first flowthrough fraction comprises MiniTrap and has a first color, wherein said first color has a b* value ranging from 1.5 to 15.0 when protein concentration is normalized to 5.0 g/L; (d) subjecting said first flowthrough fraction of step (c) to anion exchange chromatography (AEX); and (e) washing said AEX column of step (d), wherein said MiniTrap is collected in a second flowthrough fraction and has a second color, wherein said second color has a b* value ranging from 0.5 to 15.0 when protein concentration is normalized to 5.0 g/L and wherein said first color is a more intense yellow brown color than said second color."* | Original | US 2021/0206833 A1 Jul. 8, 2021  Provisional application No. 63/065,012, filed on Aug. 13, 2020.  Provisional application No. 62/944,635, filed on Dec. 6, 2019 |
| [US 11186625 B2](https://lens.org/129-037-452-994-42X) | 8/18/20 | *"1. A composition comprising oxo-aflibercept, wherein one or more amino acid residues of aflibercept is oxidized and wherein said one or more amino acid residues is histidine and/or tryptophan."* | Original | US 2021/0171605 A1 Jun. 10, 2021  Provisional application No. 63/065,012, filed on Aug. 13, 2020.  Provisional application No. 62/944,635, filed on Dec. 6, 2019. |
| [US 11174283 B2](https://lens.org/039-610-989-624-45X) | 3/18/21 | *"1. A method of producing aflibercept, comprising: (a) producing a clarified harvest of cells cultured in a chemically defined medium (CDM); (b) binding aflibercept from said clarified harvest to a Protein A resin, wherein said aflibercept includes oxidized species of aflibercept that have at least one oxidized amino acid residue selected from the group consisting of tryptophan, histidine, phenylalanine, tyrosine and a combination thereof; (c) eluting said aflibercept of step (b) forming an affinity eluate, wherein said affinity eluate comprises oxidized species of aflibercept; (d) subjecting said eluted aflibercept to anion exchange (AEX) chromatography column; and (e) collecting a flowthrough fraction, wherein the percent of oxidized species of aflibercept in said affinity eluate is greater than the percent of oxidized species of aflibercept in said flowthrough fraction when the concentrations of protein in said affinity eluate and flowthrough fraction are normalized, and wherein said oxidized species of aflibercept is measured by subjecting said affinity eluate and said flowthrough fraction to digestion, followed by their analysis using reverse-phase ultra-performance chromatography (UPLC), detection at wavelengths of 280 nm, 320 nm and 350 nm and mass spectrometry analysis using a first mobile phase of 0.1% v/v formic acid in water and a second mobile phase of 0.1% v/v formic acid in acetonitrile."* | Continuation | Continuation of application No. 16/996,007, filed on Aug. 18, 2020, now Pat. No. 11,053,280 |
| [US 11286290 B2](https://lens.org/164-502-390-616-596) | 3/24/21 | *"1. A method of purifying a VEGF MiniTrap, comprising: a) producing a clarified harvest of cells cultured in a chemically defined medium (CDM); b) binding aflibercept from said clarified harvest using an affinity chromatography column comprising a polypeptide capable of binding to or interacting with said aflibercept, wherein said polypeptide is an antibody, a fusion protein, a ScFv or a fragment thereof and wherein said polypeptide comprises an isolated amino acid sequence selected from a SEQ ID NO.: 72, SEQ ID NO.: 73, SEQ ID NO.: 74, SEQ ID NO.: 75, SEQ ID NO.: 76, SEQ ID NO.: 77, SEQ ID NO.: 78, SEQ ID NO.: 79 and SEQ ID NO.: 80; c) eluting said aflibercept of step (b) and subjecting said aflibercept to enzymatic cleavage to remove its Fc domain thereby forming a sample having a VEGF MiniTrap; optionally, d) subjecting said sample to a second chromatography capture step; and e) collecting a flowthrough fraction, wherein said flowthrough fraction has said VEGF MiniTrap."* | Continuation | Continuation of application No. 16/996,042, filed on Aug. 18, 2020, now Pat. No. 11,098,112 . |
| [US 11440950 B2](https://lens.org/130-220-018-562-510) | 7/16/21 | *"1. A recombinant polypeptide having IdeS protease activity comprising the amino acid sequence selected from the group consisting of SEQ ID NO.: 2, SEQ ID NO.: 3, SEQ ID NO.: 4, SEQ ID NO.: 5, SEQ ID NO.: 6, SEQ ID NO.: 7, SEQ ID NO.: 8, SEQ ID NO.: 9, SEQ ID NO.: 10, SEQ ID NO.: 11, SEQ ID NO.: 12, SEQ ID NO.: 13, SEQ ID NO.: 14, SEQ ID NO.: 15 and SEQ ID NO.: 16, wherein said polypeptide has the IdeS protease activity at a basic pH."* | Continuation | Continuation of application No. 16/996,103, filed on Aug. 18, 2020, now Pat. No. 11,098,311 |
| [US 11535663 B2](https://lens.org/105-490-066-972-913) | 7/22/21 | *"1. A method of producing aflibercept harvested from a host cell cultured in a chemically defined medium (CDM), comprising: a) culturing said host cell in said CDM under conditions suitable in which said host cell expresses said aflibercept, wherein the cumulative concentration of copper in said CDM is less than or equal to 0.8 μM or about 0.8 μM and the cumulative concentration of nickel in said CDM is less than or equal to 0.4 μM, and one or more of the following: i) the cumulative concentration of iron in said CDM is less than or equal to 55.0 μM; ii) the cumulative concentration of zinc in said CDM is less than or equal to 56.0 μM; iii) the cumulative concentration of cysteine in said CDM is less than or equal to 10.0 mM; and iv) said CDM includes anti-oxidants where the cumulative concentration of an antioxidant is about 0.001 mM to about 10.0 mM for any single anti-oxidant; and b) harvesting aflibercept produced by said host cell."* | Continuation | Continuation of application No. 16/996,030, filed on Aug. 18, 2020, now Pat. No. 11,104,715 |
| [US 12054532 B2](https://lens.org/158-462-072-503-683) | 8/26/21 | *"1. An oxo-aflibercept composition wherein said oxo-aflibercept has a glycosylation profile based on molar percentage selected from the group consisting of about 40% to about 50% total fucosylated glycans, about 30% to about 55% total sialylated glycans, about 2% to about 15% mannose-5, and about 60% to about 79% galactosylated glycans."*    *"7. An oxo-aflibercept composition wherein one or more amino acid residues of histidine and/or tryptophan is oxidized on the F(ab′) 2 portion of said oxo-aflibercept."*    *"12. A prefilled syringe comprising aflibercept and oxidized variants of aflibercept that have at least one oxidized amino acid residue selected from the group consisting of His86, His110, His145, His209, His95, His19, His203, Trp58, Trp138 and a combination thereof."* | Continuation | Continuation of application No. 16/996,293, filed on Aug. 18, 2020, now Pat. No. 11,186,625 |
| [US 11306135 B2](https://lens.org/169-132-170-941-959) | 8/30/21 | *"1. A composition comprising oxo-aflibercept, wherein one or more amino acid residues of aflibercept is oxidized and wherein said one or more amino acid residues is histidine."*    *"7. A composition comprising oxo-aflibercept, wherein one or more amino acid residues of aflibercept is oxidized and wherein said one or more amino acid residues is tryptophan."*    *"14. An oxo-aflibercept composition produced in a cell culture media comprising a CDM, wherein one or more amino acid residues of aflibercept is oxidized and wherein said one or more amino acid residues is histidine and/or tryptophan."* | Continuation | Continuation of application No. 16/996,293, filed on Aug. 18, 2020, now Pat. No. 11,186,625 |
| [US 11299532 B2](https://lens.org/069-394-289-526-220) | 9/29/21 | *"1. A method of producing aflibercept, comprising: (a) binding aflibercept from a clarified harvest to a Protein A resin, wherein said aflibercept is expressed by cells cultured in a chemically defined medium (CDM), and wherein said aflibercept includes variants that have at least one oxidized amino acid residue selected from the group consisting of tryptophan, histidine, phenylalanine, tyrosine and a combination thereof; (b) eluting said aflibercept of step (a) forming an affinity eluate, wherein said affinity eluate has a first color, wherein said first color is no more yellow-brown than European Color Standard BY1; (c) subjecting said affinity eluate comprising aflibercept to anion exchange chromatography (AEX) column; and (d) collecting a flowthrough fraction, wherein said flowthrough fraction has a second color, wherein said second color has a European Color Standard BY2 or greater, and wherein said first color of said affinity eluate is a more intense yellow brown color than said second color of said flowthrough fraction when said affinity eluate and flowthrough fraction protein concentrations are normalized."* | 2nd continuation | Continuation of application No. 17/205,745, filed on Mar. 18, 2021, now Pat. No. 11,174,283 , which is a continuation of application No. 16/996,007, filed on Aug. 18, 2020, now Pat. No. 11,053,280 |
| [US 11407813 B2](https://lens.org/075-469-067-698-538) | 10/1/21 | *"1. A method of producing aflibercept MiniTrap, comprising: a) producing aflibercept using cells cultured in a chemically defined medium (CDM); b) binding said aflibercept to a first capture chromatography; c) eluting said aflibercept of step b) and subjecting said aflibercept to enzymatic cleavage to remove its Fc domain thereby forming MiniTrap; d) subjecting said formed MiniTrap of step c) to a second capture chromatography, wherein said second capture chromatography step is subjected to one or more washes to obtain a first flowthrough fraction; e) subjecting said first flowthrough fraction of step d) to an anion exchange chromatography (AEX) using an AEX column; and f) washing said AEX column of step e) to obtain a second flowthrough fraction having MiniTrap, wherein said MiniTrap has an amount of glycans selected from the group consisting of 40% to 60% total fucosylated glycans, 30% to 55% total sialylated glycans, 0% to 20% mannose-5, and 70% to 95% galactosylated glycans, wherein the amount of glycans are determined using hydrophilic interaction liquid chromatography with post-column fluorescence detection, and wherein the glycans are separated and eluted using acetonitrile and aqueous 50 mM ammonium formate (pH 4.4) and detected using a fluorescence detector with an excitation wavelength of 265 nm and an emission wavelength of 425 nm."* | Continuation | Continuation of application No. 16/996,127, filed on Aug. 18, 2020, now Pat. No. 11,180,540 |
| [US 11459373 B2](https://lens.org/187-651-607-769-694) | 10/13/21 | *"1. A method of producing aflibercept, comprising: a. culturing cells which express aflibercept in a chemically defined medium (CDM), and producing a clarified harvest from the cell culture; b. binding aflibercept from said clarified harvest to a Protein A resin; c. eluting said aflibercept of step (b) forming an affinity eluate, and subjecting said eluate comprising aflibercept to anion exchange chromatography (AEX); and d. collecting a flowthrough fraction, wherein said aflibercept of step d) has a glycosylation profile based on molar percentage selected from the group consisting of about 40% to about 50% total fucosylated glycans, about 30% to about 55% total sialylated glycans, about 2% to about 15% mannose-5, and about 60% to about 79% galactosylated glycans."* | 3rd continuation | Continuation of application No. 17/489,495, filed on Sep. 29, 2021 , which is a continuation of application No. 17/205,745, filed on Mar. 18, 2021, now Pat. No. 11,174,283 , which is a continuation of application No. 16/996,007, filed on Aug. 18, 2020, now Pat. No. 11,053,280 |
| [US 11472861 B2](https://lens.org/022-206-232-086-213) | 12/21/21 | *"1. A method of producing aflibercept harvested from a host cell cultured in a chemically defined medium (CDM), comprising: (a) providing a host cell genetically engineered to express aflibercept; (b) culturing said host cell in said CDM under conditions suitable in which said host cell expresses said aflibercept wherein the cumulative concentration of cysteine in said CDM is less than or equal to 10.0 mM and the cumulative concentration of nickel in said CDM is less than or equal to 0.4 μM or about 0.4 and one or more of the following: i. the cumulative concentration of iron in said CDM is less than or equal to 55.0 μM; ii. the cumulative concentration of copper in said CDM is less than or equal to 0.8 μM; iii. the cumulative concentration of zinc in said CDM is less than or equal to 56.0 μM; and iv. said CDM includes anti-oxidants where the cumulative concentration of an antioxidant is about 0.001 mM to about 10.0 mM for any single anti-oxidant; and (c) harvesting aflibercept produced by said host cell."* | 2nd continuation | Continuation of application No. 17/383,322, filed on Jul. 22, 2021 , which is a continuation of application No. 16/996,030, filed on Aug. 18, 2020, now Pat. No. 11,104,715 |
| [US 11485770 B2](https://lens.org/142-629-846-478-436) | 1/13/22 | *"1. A culture media composition comprising chemically defined media (CDM) and oxo-aflibercept, wherein one or more amino acid residues of aflibercept is oxidized and wherein said one or more amino acid residues is histidine and/or tryptophan."* | Division | Division of application No. 17/460,578, filed on Aug. 30, 2021, now Pat. No. 11,306,135 , which is a continuation of application No. 16/996,293, filed on Aug. 18, 2020, now Pat. No. 11,186,625 |
| [US 11649273 B2](https://lens.org/173-031-708-553-344) | 1/13/22 | *"1. A method of producing aflibercept, comprising: a) producing a clarified harvest of cells cultured in a chemically defined medium (CDM); b) binding aflibercept from said clarified harvest to an affinity chromatography column comprising a polypeptide capable of binding to or interacting with said aflibercept, wherein said polypeptide is an antibody, a fusion protein, a ScFv or a fragment thereof; c) eluting said aflibercept of step (b) forming an affinity eluate, wherein said eluate has a first color; d) subjecting said eluate comprising aflibercept of step (c) to anion exchange chromatography (AEX); and e) collecting a flowthrough fraction, wherein the flowthrough fraction comprises said aflibercept, and wherein said flowthrough fraction has a second color, wherein the first color is more yellow brown than the second color."* | 2nd continuation | Continuation of application No. 17/211,333, filed on Mar. 24, 2021, now Pat. No. 11,286,290 , which is a continuation of application No. 16/996,042, filed on Aug. 18, 2020, now Pat. No. 11,098,112 |
| [US 11459374 B2](https://lens.org/027-547-342-407-96X) | 2/8/22 | *"1. A method for preparing a prefilled syringe comprising the step of loading a syringe with aflibercept, wherein a process of producing aflibercept comprises the steps of: (a) binding aflibercept produced in a chemically defined media (CDM) to a first capture chromatography, wherein said aflibercept includes oxidized species of aflibercept that have at least one oxidized amino acid residue selected from the group consisting of tryptophan, histidine, and a combination thereof; (b) eluting said aflibercept of step (a) forming an affinity eluate, wherein said eluate has a first color; (c) subjecting said affinity eluate to an ion exchange chromatography column; and (d) collecting a flowthrough fraction, wherein said flowthrough fraction has a second color, and wherein said first color is a more intense yellow-brown color than said second color when said affinity eluate and said flowthrough fraction protein concentrations are normalized to 5.0 g/L."* | 4th continuation | Continuation of application No. 17/500,735, filed on Oct. 13, 2021 , which is a continuation of application No. 17/489,495, filed on Sep. 29, 2021, now Pat. No. 11,299,532 , which is a continuation of application No. 17/205,745, filed on Mar. 18, 2021, now Pat. No. 11,174,283 , which is a continuation of application No. 16/996,007, filed on Aug. 18, 2020, now Pat. No. 11,053,280 |
| [US 11505593 B2](https://lens.org/048-763-024-260-681) | 2/8/22 | *"1. A prefilled syringe comprising aflibercept produced in chemically defined media (CDM), wherein said aflibercept includes oxidized variants that have at least one oxidized amino acid residue selected from the group consisting of tryptophan, histidine, and a combination thereof."*    *"21. A prefilled syringe comprising aflibercept having a BY value of at least 5 when the concentration of aflibercept is normalized to 5 g/L protein concentration, wherein said aflibercept includes oxidized variants that have at least one oxidized amino acid residue selected from the group consisting of tryptophan, histidine, and a combination thereof."* | Continuation | Continuation of application No. 17/575,425, filed on Jan. 13, 2022 , which is a division of application No. 17/460,578, filed on Aug. 30, 2021, now Pat. No. 11,306,135 , which is a continuation of application No. 16/996,293, filed on Aug. 18, 2020, now Pat. No. 11,186,625 |
| [US 11505594 B2](https://lens.org/048-470-911-695-316) | 4/1/22 | *"1. A method of producing MiniTrap from a host cell cultured in a chemically defined medium (CDM), comprising: (a) culturing said host cell in said CDM under suitable conditions in which said host cell expresses aflibercept wherein (i) the cumulative concentration of nickel in said CDM is less than or equal to 0.4 μM, (ii) the cumulative concentration of iron in said CDM is less than or equal to 55.0 μM, (iii) the cumulative concentration of copper in said CDM is less than or equal to 0.8 μM, (iv) the cumulative concentration of zinc in said CDM is less than or equal to 56.0 μM, (v) the cumulative concentration of cysteine in said CDM is less than or equal to 10.0 mM, or (vi) the cumulative concentration of an antioxidant in said CDM is 0.001 mM to 10.0 mM for any single anti-oxidant and the cumulative concentration of all anti-oxidants is less than or equal to 30.0 mM; (b) binding said aflibercept from said host cell cultured in said CDM to a first capture chromatography; (c) eluting said aflibercept of step (b) and subjecting said aflibercept in eluate to enzymatic cleavage to remove its Fc domain thereby forming said MiniTrap; (d) subjecting said MiniTrap of step (c) to a second capture chromatography, wherein said second capture chromatography step is subjected to one or more washes, wherein a flowthrough fraction comprises MiniTrap and has a first color, wherein said first color has a b* value ranging from 1.5 to 15.0 when protein concentration is normalized to 5.0 g/L, when characterized in the CIE L*, a*, b* color space, such that L* is about 70 to about 99, a* is about 0; (e) subjecting said flowthrough fraction of step (d) to anion exchange chromatography (AEX) with an AEX column; and (f) washing said AEX column of step (e), wherein said MiniTrap is collected in a flowthrough fraction and has a second color, wherein said second color has a b* value ranging from 0.4 to 15.0 when protein concentration is normalized to 5.0 g/L and wherein said first color is a more intense yellow brown color than said second color."* | 2nd continuation | Continuation of application No. 17/492,440, filed on Oct. 1, 2021 , which is a continuation of application No. 16/996,127, filed on Aug. 18, 2020, now Pat. No. 11,180,540 |
| [US 11548932 B2](https://lens.org/187-656-122-132-41X) | 6/15/22 | *"1. A method of producing aflibercept from a host cell cultured in a chemically defined medium (CDM), comprising: (a) culturing said host cell in said CDM under suitable conditions in which said host cell expresses aflibercept wherein (i) the cumulative concentration of nickel in said CDM is less than or equal to 0.4 μM, (ii) the cumulative concentration of iron in said CDM is less than or equal to 55.0 μM, (iii) the cumulative concentration of copper in said CDM is less than or equal to 0.8 μM, (iv) the cumulative concentration of zinc in said CDM is less than or equal to 56.0 μM, (v) the cumulative concentration of cysteine in said CDM is less than or equal to 10.0 mM, or (vi) the cumulative concentration of an antioxidant in said CDM is 0.001 mM to 10.0 mM for any single anti-oxidant and the cumulative concentration of all anti-oxidants is less than or equal to 30.0 mM; (b) binding said aflibercept from said host cell cultured in said CDM to a first capture chromatography; (c) eluting said aflibercept of step (b); (d) subjecting said aflibercept of step (c) to a second capture chromatography, wherein said second capture chromatography step is subjected to one or more washes, wherein a flowthrough fraction comprises aflibercept and has a first color, wherein said first color has a b* value ranging from 1.5 to 15.0 when protein concentration is normalized to 5.0 g/L, when characterized in the CIE L*, a*, b* color space, such that L* is about 70 to about 99, a* is about 0; (e) subjecting said flowthrough fraction of step (d) to anion exchange chromatography (AEX) with an AEX column; and (f) washing said AEX column of step (e), wherein said aflibercept is collected in a flowthrough fraction and has a second color, wherein said second color has a b* value ranging from 0.4 to 15.0 when protein concentration is normalized to 5.0 g/L and wherein said first color is a more intense yellow brown color than said second color."* | 3rd continuation | Continuation of application No. 17/711,723, filed on Apr. 1, 2022 , which is a continuation of application No. 17/492,440, filed on Oct. 1, 2021, now Pat. No. 11,407,813 , which is a continuation of application No. 16/996,127, filed on Aug. 18, 2020, now Pat. No. 11,180,540 |
| [US 11542317 B1](https://lens.org/042-775-198-645-40X) | 6/16/22 | *"1. A formulation comprising: (a) 40 to 200 mg/ml of aflibercept produced in chemically defined media (CDM), wherein said aflibercept includes oxidized variants that have at least one oxidized amino acid residue selected from the group consisting of tryptophan, histidine, and a combination thereof; (b) one or more pharmaceutically acceptable excipients selected from the group of buffering agents, preservatives, detergents and combinations thereof; and (d) said formulation having a pH between 5.0 and 8.0."*    *"20. A method of administering a pharmaceutically effective amount of aflibercept by intravitreal injection wherein said aflibercept is made in chemically defined media (CDM) and includes oxidized variants that have at least one oxidized amino acid residue selected from the group consisting of tryptophan, histidine, and a combination thereof and one or more pharmaceutically acceptable excipients."*    *"26. A method of treating Neovascular (Wet) Age-Related Macular Degeneration (AMD), Macular Edema Following Retinal Vein Occlusion (RVO), Diabetic Macular Edema (DME), or Diabetic Retinopathy with aflibercept wherein said aflibercept is made in chemically defined media (CDM) and includes oxidized variants that have at least one oxidized amino acid residue selected from the group consisting of tryptophan, histidine, and a combination thereof."* | 2nd continuation | Continuation of application No. 17/667,298, filed on Feb. 8, 2022 , which is a continuation of application No. 17/575,425, filed on Jan. 13, 2022 , which is a division of application No. 17/460,578, filed on Aug. 30, 2021, now Pat. No. 11,306,135 , which is a continuation of application No. 16/996,293, filed on Aug. 18, 2020, now Pat. No. 11,186,625 |
| [US 11753459 B2](https://lens.org/142-913-907-566-48X) | 7/6/22 | *"1. An oxo-aflibercept composition produced in a cell culture media comprising a chemically defined media (CDM), wherein one or more amino acid residues of aflibercept is oxidized and wherein said one or more amino acid residues is histidine and/or tryptophan, and wherein said oxo-aflibercept has a glycosylation profile based on molar percentage selected from the group consisting of about 40% to about 50% total fucosylated glycans, about 30% to about 55% total sialylated glycans, about 2% to about 15% mannose-5, and about 60% to about 79% galactosylated glycans."*    *"7. A culture media composition comprising chemically defined media (CDM) and oxo-aflibercept, wherein one or more amino acid residues of aflibercept is oxidized and wherein said one or more amino acid residues is histidine and/or tryptophan, and wherein said oxo-aflibercept has a glycosylation profile based on molar percentage selected from the group consisting of about 40% to about 50% total fucosylated glycans, about 30% to about 55% total sialylated glycans, about 2% to about 15% mannose-5, and about 60% to about 79% galactosylated glycans."*    *"17. A composition comprising oxo-aflibercept, wherein one or more amino acid residues of aflibercept is oxidized and wherein said one or more amino acid residues is histidine and/or tryptophan, and wherein said oxo-aflibercept has a glycosylation profile based on molar percentage selected from the group consisting of about 40% to about 52.4% total fucosylated glycans, about 30% to about 59.2% total sialylated glycans, about 5.2% to about 15% mannose-5, and about 60% to about 80.6% galactosylated glycans."* | Continuation | Continuation of application No. 17/575,425, filed on Jan. 13, 2022, now Pat. No. 11,485,770 , which is a division of application No. 17/460,578, filed on Aug. 30, 2021, now Pat. No. 11,306,135 , which is a continuation of application No. 16/996,293, filed on Aug. 18, 2020, now Pat. No. 11,186,625 . |
| [US 12054533 B2](https://lens.org/119-375-952-132-585) | 7/6/22 | *"1. A method of producing aflibercept from a host cell cultured in a chemically defined medium (CDM), comprising: (a) culturing said host cell in said CDM under suitable conditions in which said host cell expresses aflibercept; (b) purifying said aflibercept using anion exchange chromatography (AEX) and at least two of the following additional chromatography steps, in any order, selected from the group of Protein A, affinity chromatography, cation exchange chromatography (CEX), hydrophobic interaction chromatography (HIC), and mixed mode chromatography; (c) collecting said aflibercept following purification wherein said aflibercept includes between 1% and 0.03% aflibercept with at least one oxidized amino acid residue selected from the group consisting of tryptophan, histidine and a combination thereof."* | 4th continuation | Continuation of application No. 17/500,735, filed on Oct. 13, 2021, now Pat. No. 11,459,373 , which is a continuation of application No. 17/489,495, filed on Sep. 29, 2021, now Pat. No. 11,299,532 , which is a continuation of application No. 17/205,745, filed on Mar. 18, 2021, now Pat. No. 11,174,283 , which is a continuation of application No. 16/996,007, filed on Aug. 18, 2020, now Pat. No. 11,053,280 |
| [US 11732025 B2](https://lens.org/010-553-077-804-246) | 11/25/22 | *"1. A method of producing aflibercept from a host cell cultured in a chemically defined medium (CDM), comprising: (a) culturing said host cell in said CDM under suitable conditions in which said host cell expresses aflibercept wherein (i) the cumulative concentration of nickel in said CDM is less than or equal to 0.4 (ii) the cumulative concentration of iron in said CDM is less than or equal to 55.0 (iii) the cumulative concentration of copper in said CDM is less than or equal to 0.8 (iv) the cumulative concentration of zinc in said CDM is less than or equal to 56.0 (v) the cumulative concentration of cysteine in said CDM is less than or equal to 10.0 mM, or (vi) the cumulative concentration of an antioxidant in said CDM is 0.001 mM to 10.0 mM for any single anti-oxidant and the cumulative concentration of all anti-oxidants is less than or equal to 30.0 mM; (b) binding aflibercept from said clarified harvest to a Protein A resin; (c) eluting said aflibercept of step (b) forming an affinity eluate, and subjecting said eluate comprising aflibercept to anion exchange chromatography (AEX); and (d) collecting a flowthrough fraction, wherein said aflibercept of step d) has a glycosylation profile based on molar percentage selected from the group consisting of 40% to 50% total fucosylated glycans, 30% to 55% total sialylated glycans, 2% to 15% mannose-5, and 60% to 79% galactosylated glycans."* | 4th continuation | Continuation of application No. 17/841,349, filed on Jun. 15, 2022, now Pat. No. 11,548,932 , which is a continuation of application No. 17/711,723, filed on Apr. 1, 2022, now Pat. No. 11,505,594 , which is a continuation of application No. 17/492,440, filed on Oct. 1, 2021, now Pat. No. 11,407,813 , which is a continuation of application No. 16/996,127, filed on Aug. 18, 2020, now Pat. No. 11,180,540 |
| [US 12012444 B2](https://lens.org/149-366-491-932-275) | 3/1/23 | *"1. A culture media composition comprising chemically defined media (CDM) and aflibercept, wherein said aflibercept includes about 0.02% to about 1% oxidized aflibercept and wherein said oxidized aflibercept is oxidized at one or more amino acid residues selected from the group consisting of tryptophan, histidine and a combination thereof."* | 2nd continuation | Continuation of application No. 17/858,724, filed on Jul. 6, 2022 , which is a continuation of application No. 17/575,425, filed on Jan. 13, 2022, now Pat. No. 11,485,770 , which is a division of application No. 17/460,578, filed on Aug. 30, 2021, now Pat. No. 11,306,135 , which is a continuation of application No. 16/996,293, filed on Aug. 18, 2020, now Pat. No. 11,186,625 |
| [US 12077570 B2](https://lens.org/070-476-203-620-844) | 3/7/23 | *"1. A method of preparing a prefilled syringe comprising aflibercept, comprising: a) producing a clarified harvest of cells cultured in a chemically defined medium (CDM); b) binding aflibercept from said clarified harvest to an affinity chromatography column comprising a polypeptide capable of binding to or interacting with said aflibercept, wherein said polypeptide is an antibody, a fusion protein, a ScFv or a fragment thereof; c) eluting said aflibercept of step (b) forming an affinity eluate; d) subjecting said eluate comprising aflibercept of step (c) to anion exchange chromatography (AEX) column; e) collecting a flowthrough fraction, wherein the flowthrough fraction comprises said aflibercept, and wherein said aflibercept includes oxidized variants that have at least one oxidized amino acid residue selected from the group consisting of tryptophan, histidine, and a combination thereof; and f) preparing a syringe using said aflibercept of step (e) to form said prefilled syringe."* | 3rd continuation | Continuation of application No. 17/575,585, filed on Jan. 13, 2022, now Pat. No. 11,649,273 , which is a continuation of application No. 17/211,333, filed on Mar. 24, 2021, now Pat. No. 11,286,290 , which is a continuation of application No. 16/996,042, filed on Aug. 18, 2020, now Pat. No. 11,098,112 . |
| [US 11958894 B2](https://lens.org/012-494-807-387-575) | 6/23/23 | *"1. An oxo-aflibercept composition produced in a cell culture media comprising a cell culture media wherein chemically defined media (CDM) is added to cell culture media and wherein one or more tryptophan amino acid residues of aflibercept is oxidized."*    *"8. An oxo-aflibercept composition produced in a cell culture media comprising a cell culture media wherein chemically defined media (CDM) is added to cell culture media and wherein one or more amino acid residues of aflibercept is oxidized and wherein said one or more amino acid residues is histidine and/or tryptophan."*    *"13. A prefilled syringe comprising aflibercept produced in a cell culture media wherein chemically defined media (CDM) is added to cell culture media and wherein said aflibercept includes oxidized variants that have at least one oxidized amino acid residue selected from the group consisting of His86, His110, His145, His209, His95, His19, His203, Trp58, Trp138 and a combination thereof."* | 3rd continuation | Continuation of application No. 18/116,166, filed on Mar. 1, 2023 , which is a continuation of application No. 17/858,724, filed on Jul. 6, 2022 , which is a continuation of application No. 17/575,425, filed on Jan. 13, 2022, now Pat. No. 11,485,770 , which is a division of application No. 17/460,578, filed on Aug. 30, 2021, now Pat. No. 11,306,135 , which is a continuation of application No. 16/996,293, filed on Aug. 18, 2020, now Pat. No. 11,186,625 |
| [US 12049489 B2](https://lens.org/084-393-899-355-296) | 6/23/23 | *"1. A method of producing aflibercept, comprising: (a) binding aflibercept from a clarified harvest to a Protein A resin, wherein said aflibercept is expressed by cells cultured in a cell culture media wherein chemically defined media (CDM) is added to said cell culture media, and wherein said aflibercept includes variants that have at least one oxidized amino acid residue selected from the group consisting of tryptophan, histidine, phenylalanine, tyrosine and a combination thereof, (b) eluting said aflibercept of step (a) forming an affinity eluate, wherein said affinity eluate comprises oxidized species of aflibercept; (c) subjecting said eluted aflibercept to anion exchange (AEX) chromatography column; and (d) collecting a flowthrough fraction, wherein the percent of oxidized species of aflibercept in said affinity eluate is greater than the percent of oxidized species of aflibercept in said flowthrough fraction when the concentrations of protein in said affinity eluate and flowthrough fraction are normalized, and wherein said oxidized species of aflibercept is measured by subjecting said affinity eluate and said flowthrough fraction to digestion, followed by their analysis using reverse-phase ultra-performance chromatography (UPLC), detection at wavelengths of 280 nm, 320 nm and 350 nm and mass spectrometry analysis using a first mobile phase of 0.1% v/v formic acid in water and a second mobile phase of 0.1% v/v formic acid in acetonitrile."* | 5th continuation | Continuation of application No. 17/858,629, filed on Jul. 6, 2022 , which is a continuation of application No. 17/500,735, filed on Oct. 13, 2021, now Pat. No. 11,459,373 , which is a continuation of application No. 17/489,495, filed on Sep. 29, 2021, now Pat. No. 11,299,532 , which is a continuation of application No. 17/205,745, filed on Mar. 18, 2021, now Pat. No. 11,174,283 , which is a continuation of application No. 16/996,007, filed on Aug. 18, 2020, now Pat. No. 11,053,280 . |
| [US 12012445 B2](https://lens.org/064-191-542-409-81X) | 6/26/23 | *"1. A method of producing aflibercept from a host cell cultured in a cell culture media, comprising: (a) culturing said host cell in said cell culture media, wherein chemically defined media is added to said cell culture media under suitable conditions in which said host cell expresses aflibercept wherein (i) the cumulative concentration of nickel in said cell culture media is less than or equal to 0.4 μM, (ii) the cumulative concentration of iron in said cell culture media is less than or equal to 55.0 μM, (iii) the cumulative concentration of copper in said cell culture media is less than or equal to 0.8 μM, (iv) the cumulative concentration of zinc in said cell culture media is less than or equal to 56.0 μM, (v) the cumulative concentration of cysteine in said cell culture media is less than or equal to 10.0 mM, or (vi) the cumulative concentration of an antioxidant in said cell culture media is 0.001 mM to 10.0 mM for any single anti-oxidant and the cumulative concentration of all anti-oxidants is less than or equal to 30.0 mM; (b) binding said aflibercept from said host cell cultured in said cell culture media to a first capture chromatography; (c) eluting said aflibercept of step (b); (d) subjecting said aflibercept of step (c) to a second capture chromatography, wherein said second capture chromatography step is subjected to one or more washes, wherein a flowthrough fraction comprises aflibercept and has a first color, wherein said first color has a b* value ranging from 1.5 to 15.0 when protein concentration is normalized to 5.0 g/L, when characterized in the CIE L*, a*, b* color space, wherein L* is about 70 to about 99, a* is about 0; (e) subjecting said flowthrough fraction of step (d) to anion exchange chromatography (AEX) with an AEX column; and (f) washing said AEX column of step (e), wherein said aflibercept is collected in a flowthrough fraction and has a second color, wherein said second color has a b* value ranging from 0.4 to 15.0 when protein concentration is normalized to 5.0 g/L and wherein said first color is a more intense yellow brown color than said second color."* | 5th continuation | Continuation of application No. 17/994,223, filed on Nov. 25, 2022, now Pat. No. 11,732,025 , which is a continuation of application No. 17/841,349, filed on Jun. 15, 2022, now Pat. No. 11,548,932 , which is a continuation of application No. 17/711,723, filed on Apr. 1, 2022, now Pat. No. 11,505,594 , which is a continuation of application No. 17/492,440, filed on Oct. 1, 2021, now Pat. No. 11,407,813 , which is a continuation of application No. 16/996,127, filed on Aug. 18, 2020, now Pat. No. 11,180,540 |

Fig. S1. Yearly inventions of each of the nine originator biologics firms. Each chart depicts a firm’s patent families accumulated following the first application year (2004) until 2024, and shows the top listed assignees. Patent families are depicted in circles and the size of the circle reflects the number of patent members shown inside the circle. The analysis was conducted in the Orbit Intelligence platform as it allows for on site family-based analysis and the number of their automatically generated families are almost identical to those downloaded from the Lens.

From the top listed assignees in each chart, we inferred the timeline of built networks within each firm’s considered biologics collection. For example, in AbbVie’s international biologics set, the main alliances seemed limited to its own internal divisions and parent company, Abbott. Similarly, Regeneron also had very few external alliances. In contrast, Merck, Janssen, Novartis, and Sanofi consistently had a more dynamic network of alliances (partners and subsidiaries).
